# The Intrinsic Enzyme Activities of the Classic Polyoxometalates

**DOI:** 10.1038/s41598-019-50539-9

**Published:** 2019-10-16

**Authors:** Boyu Zhang, Mingming Zhao, Yanfei Qi, Rui Tian, Boye B. Carter, Hangjin Zou, Chuhan Zhang, Chunyan Wang

**Affiliations:** School of Public Health, Jilin University, Changchun, Jilin 130021 China

**Keywords:** Analytical chemistry, Inorganic chemistry

## Abstract

The mimicking enzyme activities of eighteen classic POMs with different structures, Keggin (H_3_PW_12_O_40_, H_4_SiW_12_O_40_, H_4_GeW_12_O_40_, K_4_GeW_12_O_40_, H_3_PMo_12_O_40_, H_4_SiMo_12_O_40_ and Eu_3_PMo_12_O_40_), Wells-Dawson (H_6_P_2_Mo_18_O_62_, α-(NH_4_)_6_P_2_W_18_O_62_ and α-K_6_P_2_W_18_O_62_·14H_2_O), lacunary-Keggin (Na_8_H[α-PW_9_O_34_], Na_10_[α-SiW_9_O_34_], Na_10_[α-GeW_9_O_34_] and K_8_[γ-SiW_10_O_36_]), the transition-metal substituted-type (α-1,2,3-K_6_H[SiW_9_V_3_O_34_] and H_5_PMo_10_V_2_O_40_), sandwich-type (K_10_P_2_W_18_Fe_4_(H_2_O)_2_O_68_) and an isopolyoxotungstate (Na_10_H_2_W_12_O_42_) were screened and compared. The mechanisms and reaction conditions of POMs with mimicking enzyme-like activities were also analyzed. The results shown that the structures, the hybrid atoms, the coordination atoms, the substituted metal atoms, pH and substrate are the effect factors for the enzyme mimic activities of POM. Among the eighteen POMs, H_3_PW_12_O_40_, H_4_SiW_12_O_40_, H_4_GeW_12_O_40_, α-(NH_4_)_6_P_2_W_18_O_62_, α-K_6_P_2_W_18_O_62_·14H_2_O, Na_8_H[α-PW_9_O_34_], Na_10_[α-SiW_9_O_34_], Na_10_[α-GeW_9_O_34_], K_8_[γ-SiW_10_O_36_], K_10_P_2_W_18_Fe_4_(H_2_O)_2_O_68_ and Na_10_H_2_W_12_O_42_ had the peroxidase activities. Eu_3_PMo_12_O_40_, H_3_PMo_12_O_40_, H_4_SiMo_12_O_40_, α-1,2,3-K_6_H [SiW_9_V_3_O_34_], H_6_P_2_Mo_18_O_62_ and H_5_PMo_10_V_2_O_40_ showed the oxidase-like activities. K_4_GeW_12_O_40_ did not show the peroxidase and oxidase activities. The Na_8_H[α-PW_9_O_34_], Na_10_[α-SiW_9_O_34_] and Na_10_[α-GeW_9_O_34_] showed intrinsic enzyme activities at alkaline conditions, which were different from other type of POMs. The sandwich-type K_10_P_2_W_18_Fe_4_(H_2_O)_2_O_68_ displayed the strongest peroxidase activity, which is similar to natural horseradish peroxidase.

## Introduction

Natural enzymes with high substrate specificity, activities and yields have attracted continuous scientific research interest. However, their intrinsic drawbacks, such as poor substrate versatility and assortment, low operational stabilities and low tolerance to environment conditions, limited their applications^1,2^. Therefore, artificial enzymes, as highly stable and low-cost alternatives to nature enzymes, attract continuing attention^3,4^. Constructing and screening highly efficient enzyme mimics is a tremendous motivator for researchers. To date, impressive development has been made in the field of artificial enzymes, and numerous diversity materials, such as supramolecular, porphyrins, nanoenzyme, and metal complexes have been extensively explored to mimic natural enzymes^5–9^. Generally, the efforts toward designing artificial enzymes with high activity can be divided in to two groups: the first is ‘structural mimicking’, which is to mimic the structure of enzymes, and the second is ‘functional mimicking’, which is to mimic enzymes that have similar activities^10^. Functional mimicking offers a straight forward method to discover new properties of functional materials, such as the discovery of nanoparticles with peroxidase-like activity, later referred to as nanozymes^9,11^.

Polyoxometalates (POMs), a metal oxide cluster compounds, are combinations between oxygen and early transition metals at their high oxidation states^12,13^. The majority of the applications of POMs are found in the area of catalysis^14–16^. It is reported that POMs can catalyze H_2_O_2_-based epoxidation and oxidation of organic substrates by O_2_ and H_2_O_2_ by multistep electron-transfer processes^17–19^. Therefore, it is not astonishing that POMs can used as enzyme mimics to catalyze H_2_O_2_-based oxidation of 3,3′,5,5′-tetramethylbenzidine (TMB) and Ortho-Phenylenediamine (OPD) to a colored complex which can be applied in bio- and chem-sensing, i.e., colorimetric detection of tumor cells and glucose. Wang *et al*. firstly found that folate-functionalized polyoxometalate nanoparticles have unique oxidase-like activity in colorimetric multiplexed immunoassay^20^. Moreover, they investigated the peroxidase mimetics of POMs (H_3_PW_12_O_40_, H_4_SiW_12_O_40_ and H_3_PMo_12_O_40_) and H_3_PW_12_O_40_/graphene in detection of glucose and H_2_O_2_^21^. Meanwhile, Sun *et al*. reported a simple, fast and sensitive colorimetric method to detect H_2_O_2_ based on H_4_SiW_12_O_40_^19^. Furthermore, Wang *et al*. synthesized folate-functionalized POM hybrid nanoparticles (FA-g-[(FeOH_2_)_2_SiW_10_O_36_] and FA_n_PMo_12−n_V_n_O_40_, n = 1–3) and studied the peroxidase-like activity in colorimetric assay of H_2_O_2_ and cancer cells^22^. These above early findings proved that the typical polyoxometalates and their hybrid nanoparticles have the enzyme mimics activities.

POMs with different structural morphologies can incorporated with different metal atoms. The new inorganic compositions have presented attractive enzyme mimic features. For example, Li *et al*. synthesized three new tetra-nuclear Zr^IV^- substituted POMs, which exhibit peroxidase-like activities^23^. Wang *et al*. established a colorimetric detection method based on the metal-substituted polyoxometalates of SiW_9_M_3_ (M = Co^2+^, Fe^3+^, Cu^2+^ and Mn^2+^)^24^. Xu *et al*. developed a Fe-containing heteropolyacid by cation-exchange and employed KFePW_12_O_40_ nanostructures for Fenton, photo-Fenton and enzyme-mimetic reactions^25^.

POMs can assemble with functional materials to improve their properties and potential practical applications^26^. For instance, the dipeptide-POMs-graphene oxide ternary hybrid prepared by a precipitation method show a higher peroxidase activity compared to POMs alone^27^. Inorganic-organic hybrids based on POMs and transition-metal complexes are another similar strategy to construct new enzyme mimics. Gao *et al*. synthesized and structurally characterized two new hybrids based on copper(II)-imidazole complex modified sandwich-type tungstobismuthate or tungstoantimonite, Na_4_H_2_[Cu_4_(H_4_im)_12_(H_3_im)_2_][Cu_3_(H_2_O)_3_(XW_9_O_33_)_2_] · nH_2_O (H_4_im = imidazole, H_3_im = deprotonated imidazole, X = Bi, Sb), which demonstrate higher peroxidase-like activity than Keggin-type POMs around physiological pH values in a heterogeneous phase^28^. Sha *et al*. isolated two new POM involved hybrids containing helix/nanocages ([Cu^I^_2_Cu^II^_2_(fkz)_2_(H_2_O)_7_(SiW_12_O_40_)] and [(Hfkz)_3_(H_4_SiW_12_O_40_)]) and systematically studied their peroxidase-like activities^29^. Rao *et al*. investigated the enzyme mimetic activity of a new inorganic-organic covalent hybrid of POM-calixarene^30^. Wei *et al*. reported the improved peroxidase-mimic property of the vesicles of hexavanadate-organic hybrid surfactants^31^. Metal-organic framework (MOF) based bio-sensing is a new interesting field. Pillar-layered MOFs have been proven to be an effective route to construct enzyme mimics with high stability and multifunction. The advancement of MOF structure is also help in design of new enzymes with POMs moiety. For example, Qin *et al*. report a novel efficient peroxidase mimic POM-pillared MOF, Cu_6_(Trz)_10_(H_2_O)_4_[H_2_SiW_12_O_40_] ·8H_2_O^32^. Recently, *Sha et al*. reported a stable peroxidase mimic POM-pillared metal–MOF with 6-nuclear Cu-pz and 10-nuclear Cu-pz-Cl cycles, [Cu_5_(pz)_6_Cl] [SiW_12_O_40_]^33^.

Most of the POMs are stable and show higher enzyme activities at acid condition (about pH value 3 or 4). However, in the physiological solutions (pH 7.0–7.5), for most bioanalytical applications, the POM nanozymes become catalytically inactive. Fortunately, POMs show a great diversity in its structure derived from its multiple oxidation states and coordination geometries^30,34^. These features make it much easier to control the size, shape, and charge distribution at the molecular level. Flexibility in the structure makes it possible to fine-tune the redox potentials, acidities, and enzyme activities of POMs. For example, the trivacant Keggin Na_10_[α-SiW_9_O_34_] exhibits unusual peroxidase-like activity at basic condition^35^. Therefore, it is necessary to systematic investigate of POMs mimic enzymes activity with different structure category. Herein, the mimicking enzyme activities of classic polyoxometalates with different classic structures and different element atoms were screened and compared.

## Results and Discussion

### Characterization of POMs

The POMs were prepared according to the literature and identified by FI-IR spectra, UV-Vis spectra, as shown in Supplementary Figs S1 and S2.

### Enzyme mimetic activities of POMs

**Figure 1:**
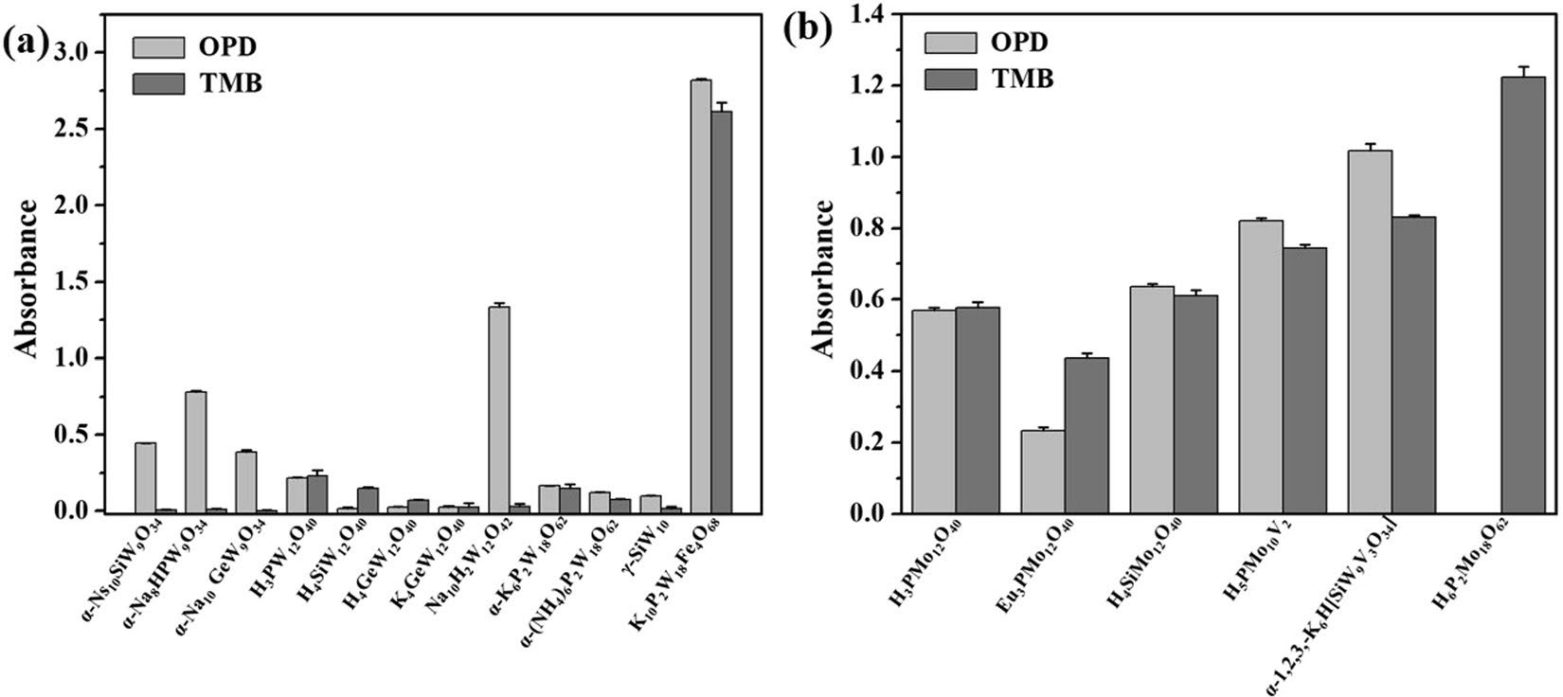
Comparison of enzyme mimic activities of polyoxometalates. (**a**) peroxidase-like activities with TMB or OPD as substrate. (**b**) oxidase-like activities with TMB or OPD as substrate. Conditions: 200 *μ*M POMs, 200 mM H_2_O_2_ at room temperature for 10 minutes in the optimum pH.

As shown in Fig. 1, the enzyme mimetic activities of 18 POMs with Keggin structures (H_3_PW_12_O_40_, H_4_SiW_12_O_40_, H_4_GeW_12_O_40_, K_4_GeW_12_O_40_, H_3_PMo_12_O_40_, H_4_SiMo_12_O_40_ and Eu_3_PMo_12_O_40_), Dawson structures (H_6_P_2_Mo_18_O_62_, α-(NH_4_)_6_P_2_W_18_O_62_ and α-K_6_P_2_W_18_O_62_·14H_2_O), lacunary-Keggin structures (Na_8_H[α-PW_9_O_34_], Na_10_[α-SiW_9_O_34_], Na_10_[α-GeW_9_O_34_] and K_8_[γ-SiW_10_O_36_]), the transition-metal substituted-type structures (α-1,2,3-K_6_H[SiW_9_V_3_O_34_], H_5_PMo_10_V_2_O_40_) and sandwich-type K_10_P_2_W_18_Fe_4_(H_2_O)_2_O_68_) were studied and compared at the same concentration with OPD and TMB as substrates. It was found that the coordination atoms, Mo and W, have effect on the enzyme mimic activity. All the polyoxotungstates H_3_PW_12_O_40_, H_4_SiW_12_O_40_, H_4_GeW_12_O_40_, K_4_GeW_12_O_40_, Na_10_[α-GeW_9_O_34_], Na_8_H[α-PW_9_O_34_], Na_10_[α-SiW_9_O_34_], K_8_[γ-SiW_10_O_36_], α-(NH_4_)_6_P_2_W_18_O_62_, α-K_6_P_2_W_18_O_62_·14H_2_O, Na_10_H_2_W_12_O_42_ and K_10_P_2_W_18_Fe_4_(H_2_O)_2_O_68_ are capable of catalyzing typical peroxidase reactions using both chromogenic hemeperoxidase substrates TMB and OPD in the presence of H_2_O_2_ to produce a blue color (maximum absorbance 650 nm) and brown color (maximum absorbance 450 nm) reaction, respectively, as shown in Fig. 1a. Initially, these reactions were carried out by adding 200 *μ*M of POMs with the substrates OPD (576 *μ*M) and H_2_O_2_ (200 mM) at room temperature in a buffered solution. It indicates that these POMs have intrinsic peroxidase-like activities towards these substrates (Fig. 1). However, the peroxidase-like activities of these POMs are different in the present of different substrates at the same concentration. For TMB as organic substrate, the absorption values of TMB^+^ indicated that the order of peroxidase-like activities from high to low was K_10_P_2_W_18_Fe_4_(H_2_O)_2_O_68_ > H_3_PW_12_O_40_ > H_4_SiW_12_O_40_ > α-K_6_P_2_W_18_O_62_·14H_2_O > α-(NH_4_)_6_P_2_W_18_O_62_ > H_4_GeW_12_O_40_ > Na_10_H_2_W_12_O_42_ > K_4_GeW_12_O_40_ > K_8_[γ-SiW_10_O_36_] > Na_8_H[α-PW_9_O_34_] ≈ Na_10_[α-SiW_9_O_34_] ≈ Na_10_[α-GeW_9_O_34_]. For OPD as organic substrate, the order of peroxidase-like activities from high to low was K_10_P_2_W_18_Fe_4_(H_2_O)_2_O_68_ > Na_10_H_2_W_12_O_42_ > Na_8_H[α-PW_9_O_34_] > Na_10_[α-SiW_9_O_34_] > Na_10_[α-GeW_9_O_34_] > H_3_PW_12_O_40_ > α-K_6_P_2_W_18_O_62_·14H_2_O > α-(NH_4_)_6_P_2_W_18_O_62_ > K_8_[γ-SiW_10_O_36_] > H_4_GeW_12_O_40_ ≈ K_4_GeW_12_O_40_ ≈ H_4_SiW_12_O_40_. From the results, the lacunary-Keggin POMs, Na_10_[α-GeW_9_O_34_], Na_8_H[α-PW_9_O_34_], Na_10_[α-SiW_9_O_34_] and Na_10_H_2_W_12_O_42_ are higher affinity to the substrate of OPD than TMB. There are tiny absorbance peaks (OD values of 0.0100, 0.0070, 0.0017 and 0.0317) can be found in the above four POMs with TMB as substrates. The result indicated that peroxidase-like activities of POMs may substrate-dependence. However, no matter which the substrate was, the sandwich-type K_10_P_2_W_18_Fe_4_(H_2_O)_2_O_68_ showed the highest peroxidase activity. Under the same substrate, it was found that the hybrid atoms had effect on the peroxidase activities of the POMs and the order was P > Si > Ge. In the same hybrid atom and TMB substrate, the peroxidase activity order is Keggin structure > Wells-Dawson > lacunary-Keggin. The reactions were also carried out in the absence of the POMs at their various suitable pH values, respectively. No significant unspecific oxidation reactions were observed [Supplementary Figs S3, S4] even after half an hour. Additional control experiments using POMs in absence of H_2_O_2_ showed that no oxidative reaction occurs. Hence, these POMs had only the peroxidase-like activities defeating many mimic enzyme peroxidases which also display oxidase-like activities. The enzymatic properties of these POMs are specificity and rarely reported in the literatures^11^.

The polyoxomolybdates (H_3_PMo_12_O_40_, H_4_SiMo_12_O_40_, Eu_3_PMo_12_O_40_, α-1,2,3,-K_6_H[SiW_9_V_3_O_34_], H_5_PMo_10_V_2_O_40_ and H_6_P_2_Mo_18_O_62_) are capable of catalyzing oxidase reactions with both substrates TMB and OPD in the absence of H_2_O_2_ to produce a blue color (maximum absorbance 650 nm) and orange color (maximum absorbance 450 nm) reaction, respectively, as shown in Fig. 1b. Furthermore, the oxidase-like activities of these POMs are similar in the present of different substrates. For TMB as organic substrate, the absorption values indicated that the order of oxidase-like activities from high to low was H_6_P_2_Mo_18_O_62_ > α-1,2,3-K_6_H[SiW_9_V_3_O_34_] > H_5_PMo_10_V_2_O_40_ > H_4_SiMo_12_O_40_ > H_3_PMo_12_O_40_ > Eu_3_PMo_12_O_40_. For OPD as substrate, the order of oxidase-like activities from high to low was α-1,2,3-K_6_H[SiW_9_V_3_O_34_] > H_5_PMo_10_V_2_O_40_ > H_4_SiMo_12_O_40_ > H_3_PMo_12_O_40_ > Eu_3_PMo_12_O_40_. In the OPD substrate, the color of H_6_P_2_Mo_18_O_62_ catalytic reaction solution became dark blue with a maximum absorption peak at 710 nm. The maximum absorption peak matched with the hybrid blue, the reduction product of H_6_P_2_Mo_18_O_62_, which covered the absorption peak of the oxide product of OPD. Therefore, only TMB was chosen as the substrate for H_6_P_2_Mo_18_O_62_. Finally, K_4_GeW_12_O_40_ did not show the peroxidase and oxidase activities.

### Effect of pH

**Figure 2:**
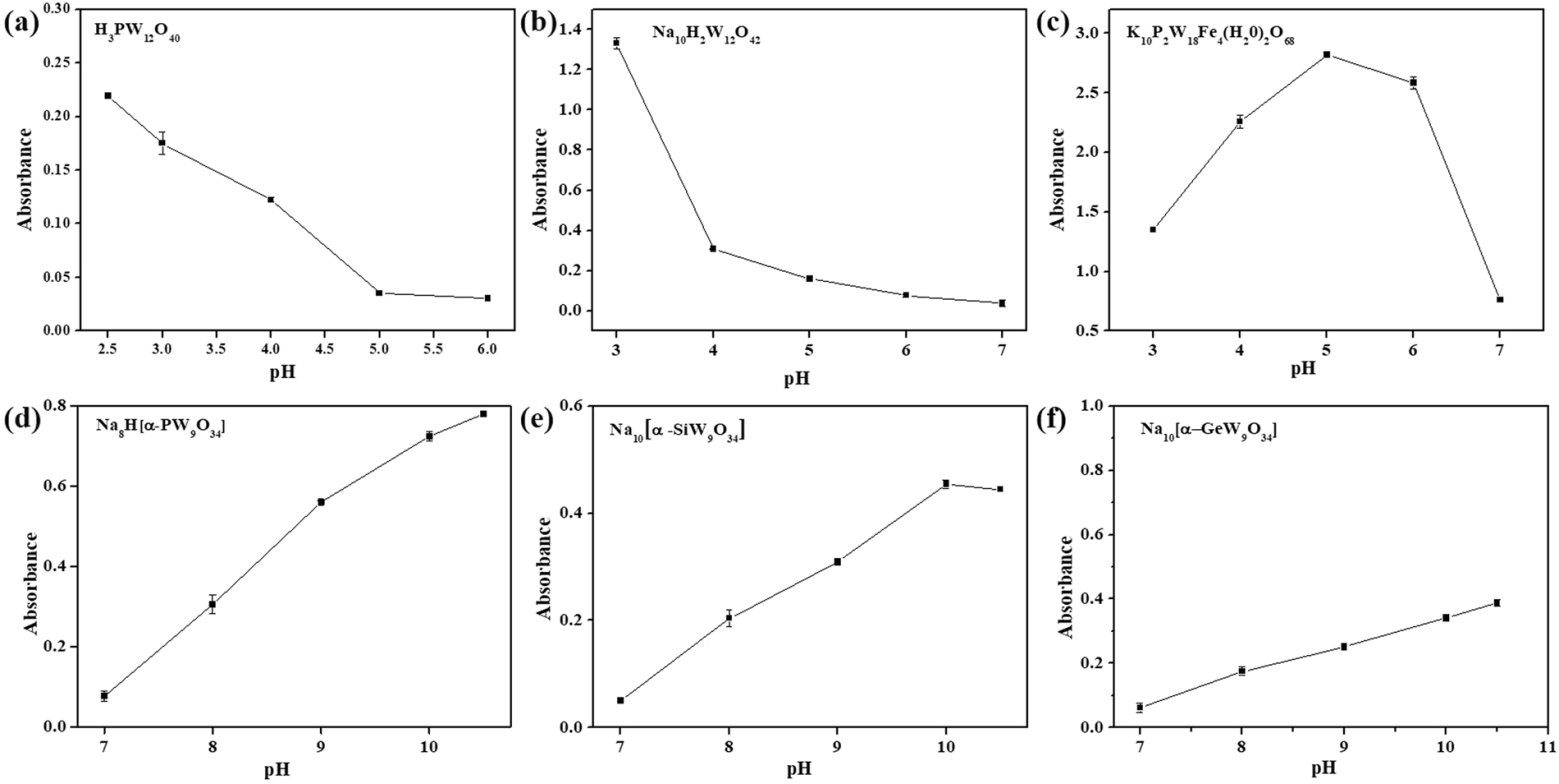
Effects of pH on peroxidase-like enzymes with OPD as a substrate, respectively. (**a**) H_3_PW_12_O_40_; (**b**)Na_10_H_2_W_12_O_42_; (**c**) K_10_P_2_W_18_Fe_4_(H_2_O)_2_O_68_; (**d**) Na_8_H[α-PW_9_O_34_]; (**e**) Na_10_[α-SiW_9_O_34_]; (**f**) Na_10_[α-GeW_9_O_34_]; Conditions: 200 *μ*M POMs, 200 mM H_2_O_2_, 0.2 mM Na_2_HPO_4_-citrate buffer for H_3_PW_12_O_40_, Na_10_H_2_W_12_O_42_ and K_10_P_2_W_18_Fe_4_(H_2_O)_2_O_68_; 0.1 mM Tris-HCl for Na_8_H[α-PW_9_O_34_], Na_10_[α-SiW_9_O_34_] and Na_10_[α-GeW_9_O_34_] at room temperature for 10 minutes. The error bars represent the standard deviation of three measurements.

The effect of pH on the catalytic activities of different types of POMs was measured by varying the pH and keeping the OPD and H_2_O_2_ concentration constant. The absorbance value of DAB with H_3_PW_12_O_40_ and Na_10_H_2_W_12_O_42_ reached a maximum at the pH 2.5, as shown in Fig. 2a,b. After exceeding this point, the absorbance decreased gradually as increasing pH values. Therefore, pH 2.5 was selected as the optimal pH value for H_3_PW_12_O_40_ and Na_10_H_2_W_12_O_42_. Similarly, pH 5 was selected as the optimal pH value for K_10_P_2_W_18_Fe_4_(H_2_O)_2_O_68_, as shown in Fig. 2c. Interestingly, the catalytic activity of the Na_10_[α-GeW_9_O_34_], Na_8_H[α-PW_9_O_34_] and Na_10_[α-SiW_9_O_34_] shows a pH optimum at alkaline conditions (~10). At this pH and in the absence of Na_10_[α-GeW_9_O_34_], Na_8_H[α-PW_9_O_34_] and Na_10_[α-SiW_9_O_34_] the unspecific reaction between OPD (576 *μ*M) and H_2_O_2_ (200 mM) was not observed. As mentioned, most of the known peroxidase-like POMs based artificial enzymes show their high activities at acid condition. Some POMs hybrids, such as inorganic-organic hybrids and FA functional particles exhibit oxidation catalyst at pH about 7.0^22,25^. However, Na_10_[α-GeW_9_O_34_], Na_8_H[α-PW_9_O_34_] and Na_10_[α-SiW_9_O_34_] are highly active even at pH above 10, as shown in Fig. 2d–f. Based on the catalytic properties of these trivacant Keggin heterotungstates, we build a CdTe quantum dots (QDs)-based fluorometric method for sensitive detection of hydrogen peroxide^35^. The effect of pH on the catalytic activities of H_3_PW_12_O_40_ and K_10_P_2_W_18_Fe_4_(H_2_O)_2_O_68_ with TMB as substrate were also investigated, as shown in Supplementary Fig. S5. The optimal pH for H_3_PW_12_O_40_ and K_10_P_2_W_18_Fe_4_(H_2_O)_2_O_68_ were 2.5 and 4, respectively.

**Figure 3:**
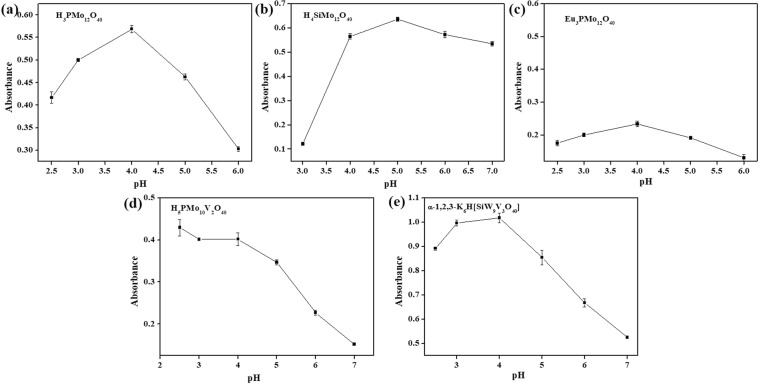
Effects of pH on oxidase-like enzymes with OPD as a substrate. (**a**) H_3_PMo_12_O_40_; (**b**) H_4_SiMo_12_O_40_; (**c**) Eu_3_PMo_12_O_40_; (**d**) H_5_PMo_10_V_2_O_40_; (**e**) α-1,2,3-K_6_H[SiW_9_V_3_O_34_]; (**f**) H_6_P_2_Mo_18_O_62_ with TMB as substrates. Conditions: 200 *μ*M POMs, 200 mM H_2_O_2_, 0.2 mM Na_2_HPO_4_-citrate buffer at room temperature for 10 minutes. The error bars represent the standard deviation of three measurements.

Analogously, the reaction pH-dependent response curves on oxidase mimics H_3_PMo_12_O_40_, H_4_SiMo_12_O_40_, Eu_3_PMo_12_O_40_, H_5_PMo_10_V_2_O_40_ and α-1,2,3-K_6_H-[SiW_9_V_3_O_34_] with OPD as substrates and H_6_P_2_Mo_18_O_62_ with TMB as substrate were shown in Fig. 3. When the pH value increased from 2.0 to 7, the absorbance reached the maximums at pH 4, 5, 3, 2.5, 4 and 5 for H_3_PMo_12_O_40_, H_4_SiMo_12_O_40_, Eu_3_PMo_12_O_40_, H_5_PMo_10_V_2_O_40_, α-1,2,3-K_6_H[SiW_9_V_3_O_34_] and H_6_P_2_Mo_18_O_62_, respectively. The effect of pH on the catalytic activities of H_3_PMo_12_O_40_, H_4_SiMo_12_O_40_, Eu_3_PMo_12_O_40_, H_5_PMo_10_V_2_O_40_ and α-1,2,3-K_6_H[SiW_9_V_3_O_34_] with TMB as substrate were also investigated, as shown in Supplementary Fig. S6. When TMB as substrates, H_3_PMo_12_O_40_, H_4_SiMo_12_O_40_, Eu_3_PMo_12_O_40_, H_5_PMo_10_V_2_O_40_ and α-1,2,3-K_6_H[SiW_9_V_3_O_34_] exhibited their strongest activities when the pH was 4, 5, 4, 2.5 and 4, respectively.

### Effect of concentration of POMs

**Figure 4:**
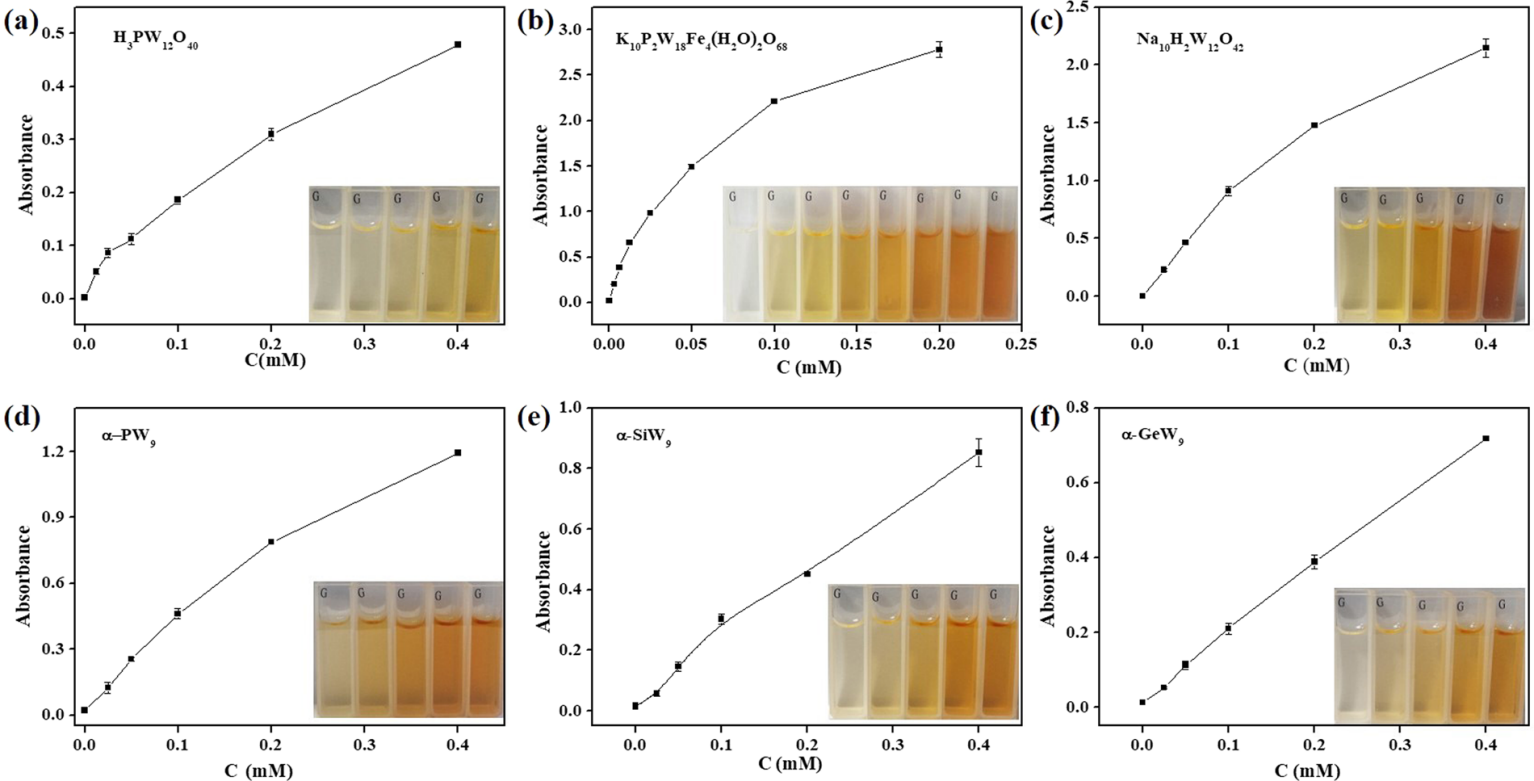
Effects of concentrations of peroxidase-like enzymes with OPD as a substrate. (**a**) H_3_PW_12_O_40_; (**b**) Na_10_H_2_W_12_O_42_; (**c**) K_10_P_2_W_18_Fe_4_(H_2_O)_2_O_68_; (**d**) Na_8_H[α-PW_9_O_34_]; (**e**) Na_10_[α-SiW_9_O_34_]; (**f**)Na_10_[α-GeW_9_O_34_]; Conditions: 200 mM H_2_O_2_, 0.2 mM Na_2_HPO_4_-citrate buffer for H_3_PW_12_O_40_, Na_10_H_2_W_12_O_42_ and K_10_P_2_W_18_Fe_4_(H_2_O)_2_O_68_; 0.1 mM Tris-HCl for Na_8_H[α-PW_9_O_34_], Na_10_[α-SiW_9_O_34_] and Na_10_[α-GeW_9_O_34_] at room temperature for 10 minutes. The error bars represent the standard deviation of three measurements.

The OPD oxidation rate catalyzed by the peroxidase-like POMs was dependent on the concentration of these POMs with the other parameters were kept constant, as shown in Fig. 4. In a typical experiment, the POMs oxidation activity was determined by monitoring of the absorbance at 450 nm (which increases because of DAB, the oxidation product of OPD, formation) for 10 min at 25 °C in different buffer for varying concentrations of POMs with respect to OPD (576 *μ*M) and H_2_O_2_ (200 mM). It can be found that the greater the amount of catalyst, the higher the yield of DAB. The increasing of the DAB yield becomes gentle when the amount of H_3_PW_12_O_40_, Na_10_H_2_W_12_O_42_, Na_10_[α-GeW_9_O_34_], Na_8_H[α-PW_9_O_34_], Na_10_[α-SiW_9_O_34_] and K_10_P_2_W_18_Fe_4_(H_2_O)_2_O_68_ exceeds 0.4, 0.4, 0.4, 0.4, 0.4 and 0.1 mM, respectively. Furthermore, Na_10_[α-GeW_9_O_34_], Na_8_H[α-PW_9_O_34_] and Na_10_[α-SiW_9_O_34_] were insoluble when the concentrations were beyond 0.4 mM. Therefore, 0.4 mM was chosen as the optimum concentrations of H_3_PW_12_O_40_, Na_10_H_2_W_12_O_42_, Na_10_[α-GeW_9_O_34_], Na_8_H[α-PW_9_O_34_] and Na_10_[α-SiW_9_O_34_] for the kinetics experiments. While, 0.1 mM was chosen for K_10_P_2_W_18_Fe_4_(H_2_O)_2_O_68_. The effect of the amounts of H_3_PW_12_O_40_ and K_10_P_2_W_18_Fe_4_(H_2_O)_2_O_68_ on peroxidase-like activities with TMB as substrates were also investigated, as shown in Supplementary Fig. S7. 0.4 mM and 0.1 mM were chosen for H_3_PW_12_O_40_ and K_10_P_2_W_18_Fe_4_(H_2_O)_2_O_68_ as the optimum concentrations, respectively.

**Figure 5:**
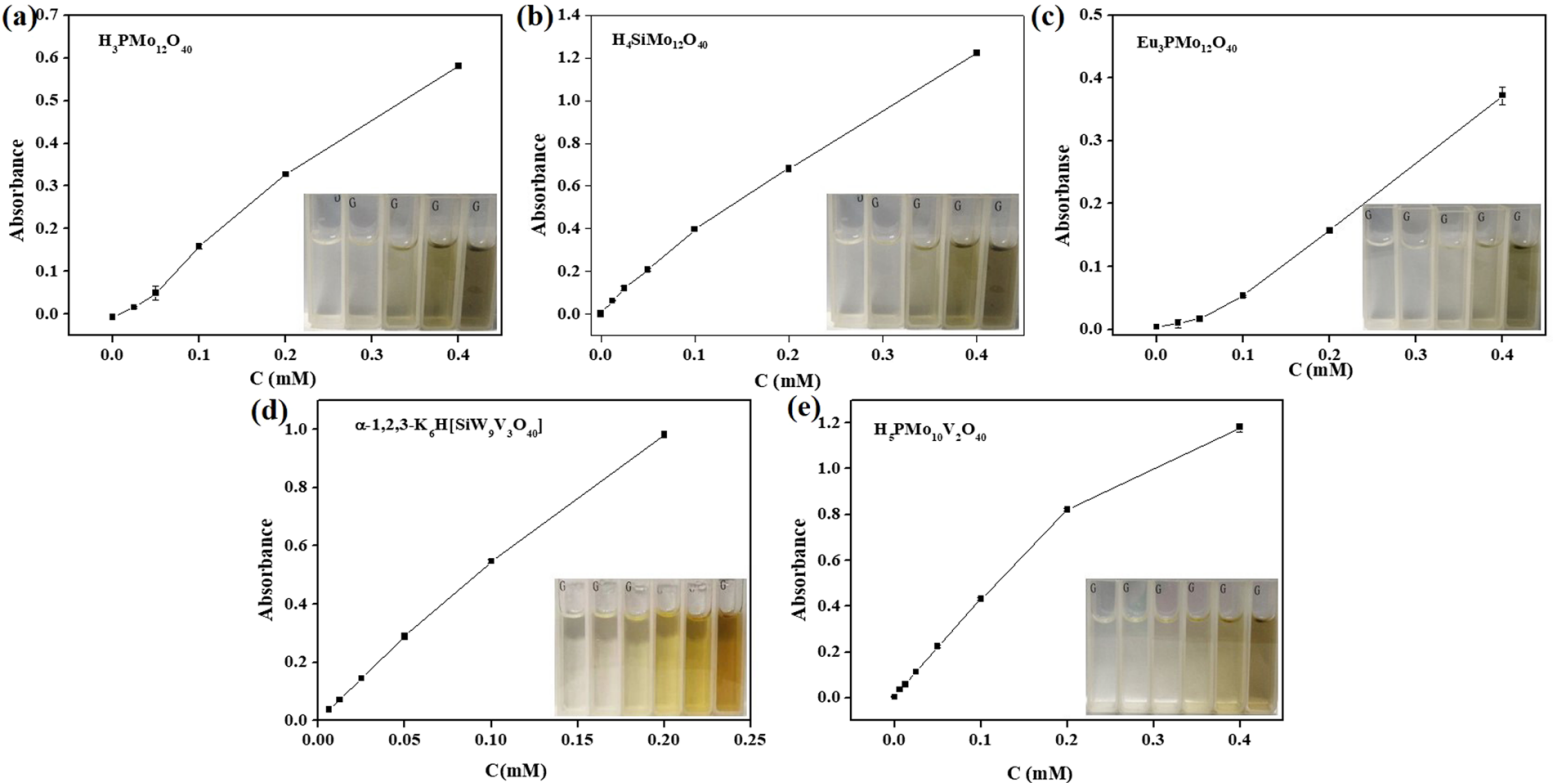
Effects of concentrations of oxidase-like enzymes with OPD as a substrate. (**a**) H_3_PMo_12_O_40_; (**b**)H_4_SiMo_12_O_40_; (**c**)Eu_3_PMo_12_O_40_; (**d**)H_5_PMo_10_V_2_O_40_; (**e**)α-1,2,3-K_6_H[SiW_9_V_3_O_34_]; (**f**)H_6_P_2_Mo_18_O_62_ with TMB as substrates. Conditions: 0.2 mM Na_2_HPO_4_-citrate buffer at room temperature for 10 minutes.The error bars represent the standard deviation of three measurements.

As shown in Fig. 5, the effect of the amounts of POMs with oxidase-like activities on the yield of DAB was also investigated. The procedures were similar with above except H_2_O_2_. DAB is obtained without H_2_O_2_, while the absorbance of DAB increases along with the increase in the amount of H_3_PMo_12_O_40_, H_4_SiMo_12_O_40_ and Eu_3_PMo_12_O_40_ and reached the maximum at 0.4 mM POMs. The maximum concentrations of H_5_PMo_10_V_2_ and α-1,2,3-K_6_H[SiW_9_V_3_O_34_] were 0.2 mM. The maximum concentration of H_6_P_2_Mo_18_O_62_ with TMB as substrates was 50 *μ*M. As shown in Fig. S8, when TMB were substrates, we found that the optimum concentration was 0.1 mM for H_3_PMo_12_O_40_, H_5_PMo_10_V_2_ and α-1,2,3- K_6_H[SiW_9_V_3_O_34_]. H_4_SiMo_12_O_40_ and Eu_3_PMo_12_O_40_ reached their maximum absorption values when the concentrations were 0.2 mM and 0.4 mM, respectively.

### Effect of reaction time

**Figure 6:**
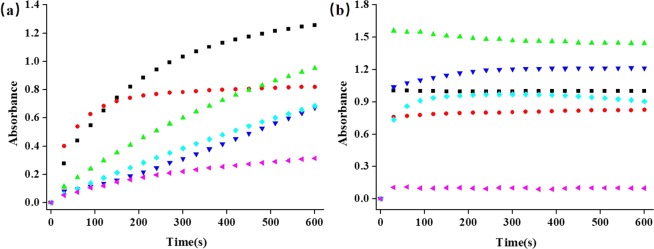
The reaction time of different-type POMs. (**a**) peroxidase (black: K_10_P_2_W_18_Fe_4_(H_2_O)_2_O_68_; red: Na_10_H_2_W_12_O_42_; green: Na_8_H[α-PW_9_O_34_]; light blue: [α-SiW_9_O_34_]; dark blue: Na_10_[α-GeW_9_O_34_]; pink: H_3_PW_12_O_40_); (**b**) oxidase (green: H_6_P_2_Mo_18_O_62_; dark blue: α-1,2,3-K_6_H[SiW_9_V_3_O_34_]; black: H_5_PMo_10_V_2_O_40_; light blue: H_4_SiMo_12_O_40_; red: H_3_PMo_12_O_40_; pink: Eu_3_PMo_12_O_40_).

The variation of the DAB yield with increasing reaction time is shown in Fig. 6. It can be observed that absorption value of DAB reaches a maximum during a reaction time of less than 10 minutes and 1 minute for peroxidase and oxidase when 200 mM H_2_O_2_ and 576 *μ*M OPD were used, respectively. After that, along with the increasing of reaction time, the yield of DAB remains nearly constant. This indicates that the optimized reaction time were 10 minutes for H_3_PW_12_O_40_, Na_10_[α-GeW_9_O_34_], Na_8_H[α-PW_9_O_34_] and Na_10_[α-SiW_9_O_34_], 5 minutes for K_10_P_2_W_18_Fe_4_(H_2_O)_2_O_68_ and 2 minutes for Na_10_H_2_W_12_O_42_, as shown in Fig. 6a. The reaction time for oxidase were less than 60 s for H_3_PMo_12_O_40_, H_4_SiMo_12_O_40_, Eu_3_PMo_12_O_40_, H_5_PMo_10_V_2_O_40_, α-1,2,3-K_6_H[SiW_9_V_3_O_34_] and H_6_P_2_Mo_18_O_62_.

### Kinetic parameters

**Figure 7:**
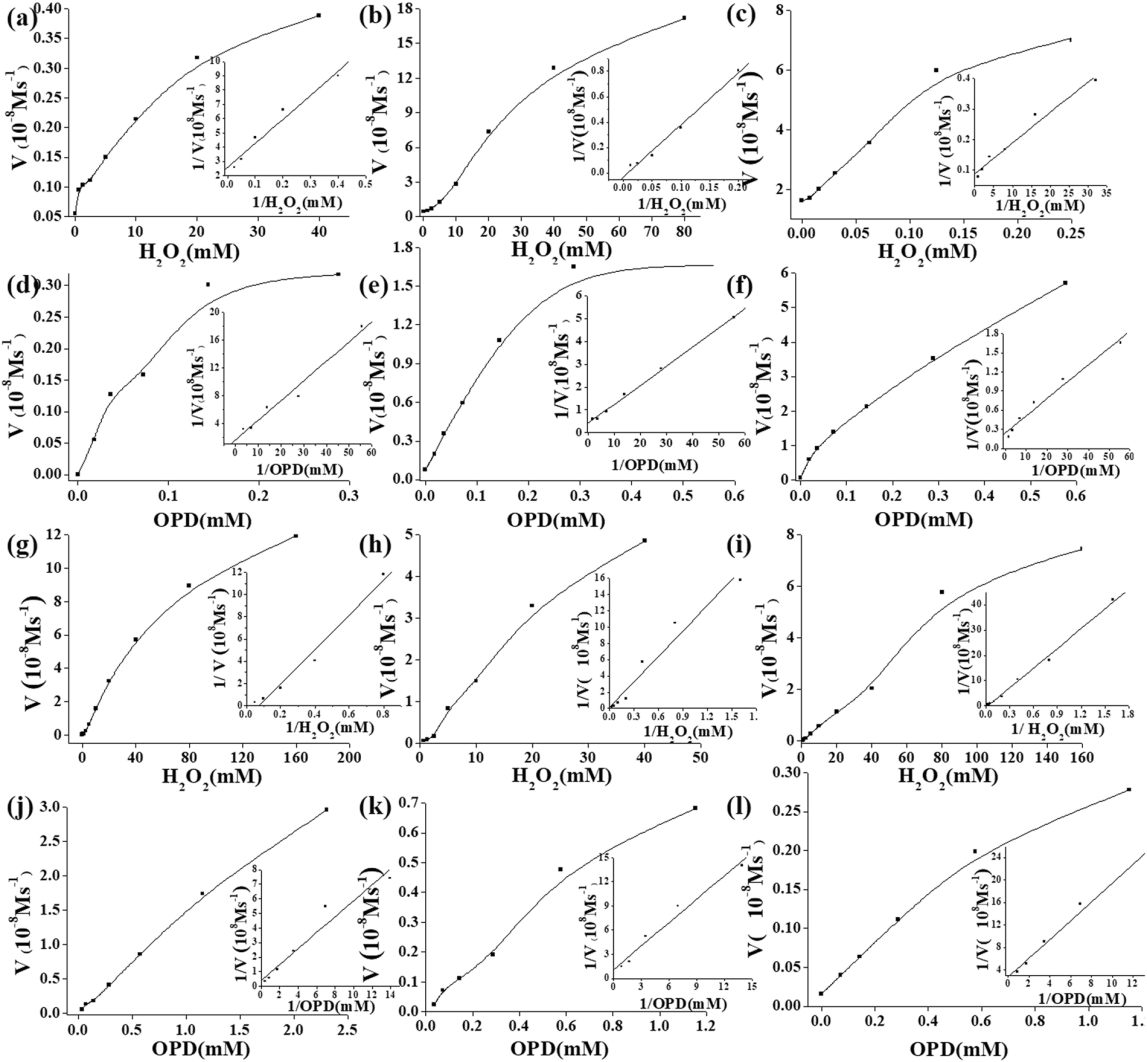
The steady-state kinetic assay and catalytic mechanism of peroxidase-like enzymes. (**a**,**b**) K_10_P_2_W_18_Fe_4_(H_2_O)_2_O_68_, (**c**,**d**) H_3_PW_12_O_40_, (**a**,**e**) Na_8_H[α-PW_9_O_34_], (**b**,**f**) Na_10_[α-SiW_9_O_34_], (**c**,**g**) Na_10_[α-GeW_9_O_34_] and (**d**,**h**) Na_10_H_2_W_12_O_42_.

**Figure 8:**
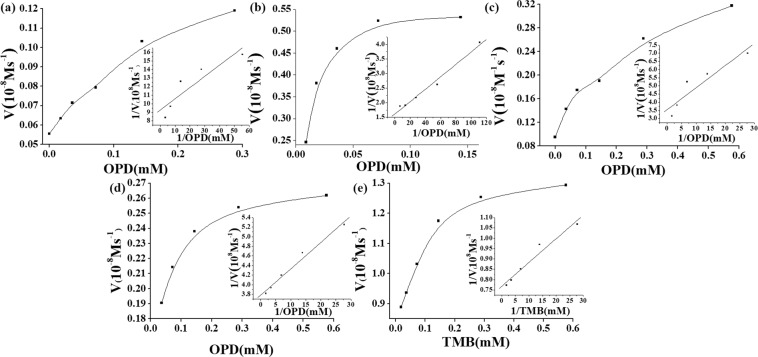
The steady-state kinetic assay and catalytic mechanism of oxidase-like enzymes. (**a**)H_3_PMo_12_O_40_, (**b**) H_4_SiMo_12_O_40_, (**c**) Eu_3_PMo_12_O_40_, (**d**) H_5_PMo_10_V_2_O_40_, (**e**) α-1,2,3-K_6_H[SiW_9_V_3_O_34_], (**f**) H_6_P_2_Mo_18_O_62_.

The mechanism of peroxidase-like catalytic activities of K_10_P_2_W_18_Fe_4_(H_2_O)_2_O_68_, H_3_PW_12_O_40_, H_4_SiW_12_O_40_, Na_10_[α-GeW_9_O_34_], Na_8_H[α-PW_9_O_34_], Na_10_[α-SiW_9_O_34_] and Na_10_H_2_W_12_O_42_ was further investigated using steady-state kinetics. It has been observed that OPD oxidation catalysis mediated by these POMs is dependent on the substrate concentration. In order to study activities of POMs, several experiments were performed whereby the concentration of either OPD or H_2_O_2_ was varied while keeping the other concentration constant. The concentration of the POMs was kept constant in all these experiments. The oxidation reaction catalyzed by K_10_P_2_W_18_Fe_4_(H_2_O)_2_O_68_, H_3_PW_12_O_40_, H_4_SiW_12_O_40_, Na_10_[α-GeW_9_O_34_], Na_8_H[α-PW_9_O_34_], Na_10_[α-SiW_9_O_34_] and Na_10_H_2_W_12_O_42_ follows a Michaelis-Menten behavior. The *K*_*m*_ and *V*_*max*_ of K_10_P_2_W_18_Fe_4_(H_2_O)_2_O_68_ and H_3_PW_12_O_40_ were obtained using a Line weaver-Burk plot with OPD as substrates, as shown in Fig. 7. The *K*_*m*_ and *V*_*max*_ of K_10_P_2_W_18_Fe_4_(H_2_O)_2_O_68_ with H_2_O_2_ as substrates were 0.113 mM and 1.13 × 10^−9^ M·S^−1^ in OPD system, as shown in Fig. 7a. The *K*_*m*_ and *V*_*max*_ of K_10_P_2_W_18_Fe_4_(H_2_O)_2_O_68_ with OPD as substrates were 0.109 mM and 4.11 × 10^−8^ M·S^−1^, as shown in Fig. 7b. The *K*_*cat*_ of K_10_P_2_W_18_Fe_4_(H_2_O)_2_O_68_ with H_2_O_2_ and OPD are 3.22 × 10^−3^ S^−1^ and 3.80 × 10^−3^ S^−1^. The *K*_*m*_ and *V*_*max*_ of H_3_PW_12_O_40_ with H_2_O_2_ as substrates were 6.624 mM and 3.89 × 10^−9^ M·S^−1^ in OPD system, as shown in Fig. 7c. The *K*_*m*_ and *V*_*max*_ of H_3_PW_12_O_40_ with OPD as substrates were 0.174 mM and 6.14 × 10^−9^ M·S^−1^, as shown in Fig. 7d. The *K*_*cat*_ of H_3_PW_12_O_40_ with H_2_O_2_ and OPD are 8.22 × 10^−4^ S^−1^ and 2.83 × 10^−6^ S^−1^. As shown in Fig. 8, the values for *K*_*m*_ OPD of Na_8_H[α-PW_9_O_34_], Na_10_[α-SiW_9_O_34_], Na_10_[α-GeW_9_O_34_] and Na_10_H_2_W_12_O_42_ were 1.39, 0.854, 0.611 and 0.205 mM, respectively. The values for *V*_*max*_ OPD of Na_8_H[α-PW_9_O_34_], Na_10_[α-SiW_9_O_34_], Na_10_[α-GeW_9_O_34_] and Na_10_H_2_W_12_O_42_ were 2.53 × 10^−8^, 8.79 × 10^−9^, 3.65 × 10^−9^ and 2.43 × 10^−8^ M·S^−1^, respectively. The *K*_*cat*_ OPD of Na_8_H[α-PW_9_O_34_], Na_10_[α-SiW_9_O_34_], Na_10_[α-GeW_9_O_34_] and Na_10_H_2_W_12_O_42_ were 4.912 × 10^−4^ S^−1^, 2.02 × 10^−4^ S^−1^, 7.03 × 10^−4^ S^−1^ and 1.46 × 10^−4^ S^−1^. The values for *K*_*m*_ H_2_O_2_ of Na_8_H[α-PW_9_O_34_], Na_10_[α-SiW_9_O_34_], Na_10_[α-GeW_9_O_34_] and Na_10_H_2_W_12_O_42_ were 13.81, 63.55, 44.05, and 132.03 mM, respectively. The *V*_*max*_ H_2_O_2_ of Na_8_H[α-PW_9_O_34_], Na_10_[α-SiW_9_O_34_], Na_10_[α-GeW_9_O_34_] and Na_10_H_2_W_12_O_42_ were 8.19 × 10^−9^, 6.07 × 10^−8^, 1.69 × 10^−8^ and 3.22 × 10^−7^ M·S^−1^, respectively. The values for *K*_*cat*_ H_2_O_2_ of Na_8_H[α-PW_9_O_34_], Na_10_[α-SiW_9_O_34_], Na_10_[α-GeW_9_O_34_] and Na_10_H_2_W_12_O_42_ were 9.73 × 10^−6^ S^−1^, 2.48 × 10^−5^ S^−1^, 1.58 × 10^−4^ S^−1^ and 4.26 × 10^−5^ S^−1^, respectively. The comparison of *K*_*m*_ and *V*_*max*_ of peroxidase-like enzymes were listed in Table 1. It was found that K_10_P_2_W_18_Fe_4_(H_2_O)_2_O_68_ had lower *K*_*m*_ than other POMs-based peroxidases with OPD or H_2_O_2_ as substrates. It suggests that K_10_P_2_W_18_Fe_4_(H_2_O)_2_O_68_ might have a similar affinity for the surfaces of OPD with HRP. The mechanism of steady-state kinetics of K_10_P_2_W_18_Fe_4_(H_2_O)_2_O_68_ were also investigated with TMB as substrates, as shown in Supplementary Fig. S8. The values for *K*_*m*_ and *V*_*max*_ TMB was 0.25 mM and 2.42 × 10^−7^ M·S^−1^, respectively. The values for *K*_*m*_ and *V*_*max*_ H_2_O_2_ was 1.09 mM and 1.02 × 10^−7^ M·S^−1^. It suggests that K_10_P_2_W_18_Fe_4_(H_2_O)_2_O_68_ might have a similar affinity for the surfaces of TMB with HRP. The *K*_*cat*_ of K_10_P_2_W_18_Fe_4_(H_2_O)_2_O_68_ with H_2_O_2_ and TMB are 4.54 × 10^−2^ S^−1^ and 8.82 × 10^−3^ S^−1^. Comparing with the other organic-inorganic POM-based hybrids as shown in Table S1, the K_10_P_2_W_18_Fe_4_(H_2_O)_2_O_68_ also show strong peroxidase activities with TMB as a substrate. It can be selected to construct new materials with organic groups or different nanomaterials.

**Table 1:** Comparison of the *K*_*m*_ and *V*_*max*_ of peroxidase-like enzymes.

Nanozymes	Substrate	*K*_*m*_ (mM)	*V*_*max*_ (M·S^−1^)
Na_8_H[α-PW_9_O_34_]	OPD	1.39	6.14 × 10^−9^
Na_8_H[α-PW_9_O_34_]	H_2_O_2_	13.81	3.89 × 10^−9^
Na_10_[α-SiW_9_O_34_]	OPD	0.85	2.53 × 10^−8^
Na_10_[α-SiW_9_O_34_]	H_2_O_2_	63.55	8.19 × 10^−9^
Na_10_[α-GeW_9_O_34]_	OPD	0.61	8.79 × 10^−9^
Na_10_[α-GeW_9_O_34_]	H_2_O_2_	44.05	6.07 × 10^−8^
Na_10_H_2_W_12_O_42_	OPD	0.2	3.65 × 10^−9^
Na_10_H_2_W_12_O_42_	H_2_O_2_	132.03	1.69 × 10^−8^
K_10_P_2_W_18_Fe_4_(H_2_O)_2_O_68_	OPD	0.10	2.43 × 10^−8^
K_10_P_2_W_18_Fe_4_(H_2_O)_2_O_68_	H_2_O_2_	0.11	3.22 × 10^−7^
H_3_PW_12_O_40_	OPD	0.17	4.11 × 10^−8^
H_3_PW_12_O_40_	H_2_O_2_	6.62	1.13 × 10^−9^
HRP	OPD	0.59	4.65 × 10^−8^ ^46^
HRP	H_2_O_2_	0.34	9.48 × 10^−8^ ^46^

The *K*_*m*_ and *V*_*max*_ were obtained for oxidase-like enzymes, as shown in Fig. 8. With OPD as substrates, the values of *K*_*m*_ OPD were 0.014, 0.013, 0.038, 0.015 and 0.015 mM for H_3_PMo_12_O_40_, H_4_SiMo_12_O_40_, Eu_3_PMo_12_O_40_, H_5_PMo_10_V_2_O_40_ and α-1,2,3, −K_6_H[SiW_9_V_3_O_34_]. The *V*_*max*_ OPD of H_3_PMo_12_O_40_, H_4_SiMo_12_O_40_, Eu_3_PMo_12_O_40_, H_5_PMo_10_V_2_O_40_ and α-1,2,3, -K_6_H[SiW_9_V_3_O_34_] were 1.08 × 10^−9^, 6.19 × 10^−9^, 2.83 × 10^−9^, 2.64 × 10^−9^ and 1.30 × 10^−8^ M·S^−1^, respectively. The *K*_*cat*_ OPD of H_3_PMo_12_O_40_, H_4_SiMo_12_O_40_, Eu_3_PMo_12_O_40_, H_5_PMo_10_V_2_O_40_ and α-1,2,3-K_6_H[SiW_9_V_3_O_34_] were 4.32 × 10^−5^, 3.09 × 10^−4^, 5.66 × 10^−5^, 1.32 × 10^−4^ and 1.73 × 10^−3^S^−1^, respectively. The comparison of *K*_*m*_ and *V*_*max*_ with different substrates were shown in Table 2. The value results of *K*_*m*_ OPD suggested that OPD have similar affinity for the surface of the oxidase-like POMs except the Eu_3_PMo_12_O_40_. α-1,2,3-K_6_H[SiW_9_V_3_O_34_] has the fastest catalytic rate than the other POMs. Among the saturated Keggin-type POMs, the counter ion Eu^3+^ maybe inhibit the poly anions interactions with the organic component. The *K*_*m*_ and *V*_*max*_ were also obtained with TMB as substrate, as shown in Supplementary Fig. S9. The *K*_*m*_ of oxidase-like enzymes was dramatically lower than natural enzyme HRP With TMB as substrates, the *K*_*m*_ were 0.616, 0.49, 0.086, 0.55, 0.02, 0.75 mM for H_3_PMo_12_O_40_, H_4_SiMo_12_O_40_, Eu_3_PMo_12_O_40_, H_5_PMo_10_V_2_O_40,_ α-1,2,3-K_6_H[SiW_9_V_3_O_34_] and H_6_P_2_Mo_18_O_62_. The *V*_*max*_ of H_3_PMo_12_O_40_, H_4_SiMo_12_O_40_, Eu_3_PMo_12_O_40_, H_5_PMo_10_V_2_O_40_, α-1,2,3-K_6_H[SiW_9_V_3_O_34_] and H_6_P_2_Mo_18_O_62_ were 2.25 × 10^−7^, 2.25 × 10^−7^, 2.60 × 10^−8^, 2.30 × 10^−7^, 1.49 × 10^−8^, 5.30 × 10^−7^ M·S^−1^, respectively. The *K*_*cat*_ of H_3_PMo_12_O_40_, H_4_SiMo_12_O_40_, Eu_3_PMo_12_O_40_, H_5_PMo_10_V_2_O_40_, α-1,2,3-K_6_H[SiW_9_V_3_O_34_] and H_6_P_2_Mo_18_O_62_ were 9.00 × 10^−3^, 1.13 × 10^−2^, 5.20 × 10^−4^, 1.15 × 10^−2^, 1.98 × 10^−3^ and 2.12 × 10^−7^S^−1^, respectively.

**Table 2:** Comparison of the *K*_*m*_ and *V*_*max*_ of oxidase-like enzymes.

Nanozymes	Substrate	*K*_*m*_ (mM)	*V*_*max*_ (M·S^−1^)	Reference
H_3_PMo_12_O_40_	OPD	0.014	1.08 × 10^−9^	This work
H_3_PMo_12_O_40_	TMB	0.625	2.25 × 10^−7^	This work
H_4_SiMo_12_O_40_	OPD	0.013	6.19 × 10^−9^	This work
H_4_SiMo_12_O_40_	TMB	0.522	2.25 × 10^−7^	This work
Eu_3_PMo_12_O_40_	OPD	0.038	2.83 × 10^−9^	This work
Eu_3_PMo_12_O_40_	TMB	0.086	2.60 × 10^−8^	This work
H_5_PMo_10_V_2_O_40_	OPD	0.015	2.64 × 10^−9^	This work
H_5_PMo_10_V_2_O_40_	TMB	0.553	2.30 × 10^−7^	This work
H_6_P_2_Mo_18_O_62_	TMB	0.750	5.30 × 10^−7^	This work
α-1,2,3-K_6_H[SiW_9_V_3_O_34_]	OPD	0.015	1.30 × 10^−8^	This work
α-1,2,3-K_6_H[SiW_9_V_3_O_34_]	TMB	0.020	1.49 × 10^−8^	This work
PMV-FA	TMB	0.00041	4.70 × 10^−6^	^20^
FAPMoV_1_	TMB	2.6 × 10^−3^	1.33 × 10^−6^	^47^
FAPMoV_3_	TMB	0.32 × 10^−3^	1.46 × 10^−5^	^47^

### Detection of the reactive hydroxyl radicals ·OH production

The mechanism of peroxidase-like activity of POMs originates from their catalytic ability to the decomposition of H_2_O_2_ to produce hydroxyl radical (·OH). Terephthalic acid (TA) can capture ·OH to generate 2-hydroxy terephthalic acid (HTA) which can be detected by fluorescence method. So we chose TA as fluorescence probe to detect the generation of ·OH. As show in Supplementary Fig. S10, the control groups (TA, TA with H_2_O_2_ and TA with POMs) did not show the significant intensity for HTA. Only in the presence of POMs and H_2_O_2_, the fluorescence can be remarkable found. This supported that the POMs can catalyze the H_2_O_2_ to generate ·OH, then justify the peroxidase-like activities of POMs.

### Calibration curve for H_2_O_2_ detection

**Figure 9:**
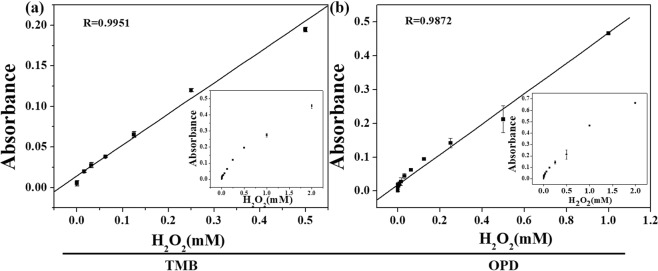
A does-response curve for K_10_P_2_W_18_Fe_4_(H_2_O)_2_O_68_ depending of the absorbance at 450 nm with OPD as substrates and at 650 nm with TMB as substrates in the presence of diverse concentrations of H_2_O_2_. (**a**)TMB as substrates; (**b**) OPD as substrates.

Because the K_10_P_2_W_18_Fe_4_(H_2_O)_2_O_68_ has the strongest the peroxidase mimetic activity, it can be used in the sensing for H_2_O_2_. Under optimum conditions, a colorimetric assay for the detection of hydrogen peroxide based on the peroxidase-like activity of K_10_P_2_W_18_Fe_4_(H_2_O)_2_O_68_ has been established with TMB and OPD as substrates, as shown in Fig. 9. When the substrate was TMB, the linear detection range was estimated to be from 15 to 1000 *μ*M with correlation coefficients 0.9951. The lower limit of detection (LOD) of K_10_P_2_W_18_Fe_4_(H_2_O)_2_O_68_ was 10.43 *μ*M. When the substrate was OPD, the correlation between the absorption value and H_2_O_2_ concentration were linear over the range of 15–500 *μ*M with correlation coefficients 0.9872. The lower limit of detection (LOD) of K_10_P_2_W_18_Fe_4_(H_2_O)_2_O_68_ was 8.86 *μ*M. In addition, the analytical performance of K_10_P_2_W_18_Fe_4_(H_2_O)_2_O_68_ as peroxidase mimics was compared with others reported POM, HRP and nanozymes, as summarized in Table 3. By comparing with other enzyme mimics, it revealed that the K_10_P_2_W_18_Fe_4_(H_2_O)_2_O_68_ sensor has wider linear range.

**Table 3:** Comparison of different nanozymes for the detection of H_2_O_2_.

Nanozymes	Linear Range	Limit of Detection	Time	Reference
K_10_P_2_W_18_Fe_4_(H_2_O)_2_O_68_	15–1000 *μ*M	10.43 *μ*M	5 min	This work^a^
K_10_P_2_W_18_Fe_4_(H_2_O)_2_O_68_	15–500 *μ*M	8.86 *μ*M	5 min	This work^b^
Cu_6_(Trz)_10_(H_2_O)_4_[H_2_SiW_12_O_40_]	10–60 *μ*M	1.37 *μ*M	1 min	^32^
Na_4_H_2_[Cu_4_(im)_14_][Cu_3_(H_2_O)_3_(BiW_9_O_33_)_2_]	1–50 *μ*M	0.12 *μ*M	2 min	^28^
Na_4_H_2_[Cu_4_(im)_14_][Cu_3_(H_2_O)_3_(SbW_9_O_33_)_2_]	1–50 *μ*M	0.12 *μ*M	2 min	^28^
FA-Fe_2_SiW_10_	0.13–67 *μ*M	0.13 *μ*M	1 min	^22^
FF@PW_12_	1–75 *μ*M	0.11 *μ*M	10 min	^27^
Na_4_(NH_4_)_14_[Zr_4_O_6_(OAc)_2_(P_2_W_16_O_59_)_2_]·51H_2_O	100–1000 *μ*M	100 *μ*M	90 min	^23^
H_4_SiW_12_O_40_	1–20 *μ*M	0.4 *μ*M	5 min	^19^
H_3_PW_12_O_40_	0.1–67 *μ*M	0.13 *μ*M	10 min	^21^
HRP	1–60 *μ*M	1 *μ*M	—	^48^
Fe_3_O_4_ MNPs	1–100 *μ*M	0.5 *μ*M	—	^49^

^a^TMB as the substrate; ^b^OPD as the substrate.

## Conclusion

In conclusion, the structures, the hybrid atoms, the coordination atoms, the substituted metal atoms are the effect factors for their enzyme mimic activities. The polyoxomolybdates (H_3_PMo_12_O_40_, H_4_SiMo_12_O_40_ and Eu_3_PMo_12_O_40_) have shown oxidase-like enzymes activities, while the polyoxotungstates shows peroxidase mimic activities. The substituted metals improved the enzyme mimic activities of POMs, which proved in H_5_PMo_10_V_2_O_40_, α-1,2,3-K_6_H[SiW_9_V_3_O_34_] and K_10_P_2_W_18_Fe_4_(H_2_O)_2_O_68_. The affinity of POM with substrate is another important factor on their enzyme mimic activities. For example, the Na_10_H_2_W_12_O_42_, Na_8_H[α-PW_9_O_34_], Na_10_[α-SiW_9_O_34_] and Na_10_[α-GeW_9_O_34_] possessed peroxidase-like activities only with OPD as the substrates. pH condition is the key impact factor not only in the stability of POM but also the enzymes activities of them. The lacunary-Keggin type POMs, Na_10_[α-GeW_9_O_34_], Na_8_H[α-PW_9_O_34_] and Na_10_[α-SiW_9_O_34_] were firstly found to own strong catalytic activities under alkaline conditions. Interesting, K_10_P_2_W_18_Fe_4_(H_2_O)_2_O_68_ showed the highest catalytic activities among the POMs. In the future, the further analysis the inorganic systems and functionalization of metal-substituted polyoxotungstates will be expected to have a potential application in biotechnology, clinical diagnosis and other industry to substitute natural enzymes.

## Experimental

### Chemicals and materials

All the chemicals used were of analysis and graded without further purification. OPD was purchased from Tianjin Guangfu Fine Chemical Research Institute (Tianjin, China). TMB was obtained from (Tokyo, Japan). H_3_PW_12_O_40_, H_4_SiW_12_O_40_ and H_3_PMo_12_O_40_ and Hydrogen peroxide (H_2_O_2_, 30%) were purchased from Beijing Chemical Works (Beijing, China). The water used in the experiments was purified. The POMs mimetic enzymes were characterized by IR and UV-vis spectrum. The UV-*vis* spectrum was recorded in the range of 200–600 nm on UV-Vis spectrophotometer (Puxi Inc., Beijing, China). Fourier-transform infrared spectrum (FT-IR) was collected in the range of 4000–400 cm^−1^ on an Alpha Centauri FT/IR spectrophotometer (Shizumi, Tokyo) using KBr pellets.

### Synthesis of polyoxometalates

The 18 polyoxometalates including Keggin (H_3_PW_12_O_40_, H_4_SiW_12_O_40_, H_4_GeW_12_O_40_, K_4_GeW_12_O_40_, H_4_SiMo_12_O_40_, H_3_PMo_12_O_40_, Eu_3_PMo_12_O_40_), Dawson (H_6_P_2_Mo_18_O_62_, α-(NH_4_)_6_P_2_W_18_O_62_, α-K_6_P_2_W_18_O_62_·14H_2_O), lacunary-Keggin (Na_10_[α-GeW_9_O_34_], Na_8_H[α-PW_9_O_34_], Na_10_[α-SiW_9_O_34_], K_8_[γ-SiW_10_O_36_]) and the transition-metal substituted-type (α-1,2,3-K_6_H[SiW_9_V_3_O_34_], H_5_PMo_10_V_2_O_40_, Wells-Dawson (K_10_P_2_W_18_Fe_4_(H_2_O)_2_O_68_) investigated in this study were synthesized according to the literature^36–43^ or provide by prof. Yangguang Li.

### Enzyme mimetic activities of POMs

The enzyme mimetic activities of POMs were determined spectrophotometrically by measuring the formation of DAB from OPD at 450 nm (ε = 2.1 × 10^4^ mM^−1^ cm^−1^)^44^ or TMB^+^ from TMB at 650 nm (ε = 3.9 × 10^4^ mM^−1^ cm^−1^)^45^ using UV-vis spectrophotometer in a 1 cm cuvette. Typically, the 480 *μ*L of TMB (1.5 mM in ethanol) or OPD solution (3.6 mM in water) was added into 2400 *μ*L different buffer solutions (NaH_2_PO_4_-citrate or Tris-HCl), followed by the addition of 60 *μ*L of POMs (10 mM) and 60 *μ*L of hydrogen peroxide (H_2_O_2_, 10 M). The mixed solution was incubated at room temperature. The oxidases activities of POMs (10 mM) were used under the same identical reaction conditions with the absence of H_2_O_2_.

### pH measurements

The activities of the POMs at different pH values were performed using the same condition as above, except two different buffer compositions for the different pH ranges were employed. The reaction was carried out 200 μM POMs to which TMB (240 μM) or OPD (576 *μ*M) and H_2_O_2_ (200 mM) were added. Between pH 2.5 to 7, 0.2 mM of Na_2_HPO_4_-citrate buffer was used; for pH 7–10, 0.1 mM of Tris-HCl buffer was used. The pH of the different buffers was adjusted using a pH meter (PHS-25 pH meter, Shanghai INESA Scientific Instrument Co., China).

### Determination of kinetic parameters

The steady-state kinetics of POM-peroxidase were conducted by varying the concentrations of H_2_O_2_ (0–200 mM), or OPD/TMB (0–576 *μ*M/0–240 *μ*M) one at a time. The reaction was carried out in 0.2 mM Na_2_HPO_4_-citrate buffer and 0.1 mM Tris-HCl (at the optimum pH) and monitored spectrophotometrically by 300 s using a 1 cm cuvette. The kinetic curves were adjusted to the Michaelis-Menten model and linear weaver-Burk linearizations were performed using origin 7.0 software. The apparent kinetic parameters were calculated based on the equation *v* = *V*_*max*_ × [S]/(*K*_*m*_ + [S]), where *v* is the initial velocity, *V*_*max*_ is the maximal reaction velocity, [S] is the concentration of substrate, and *K*_*m*_ is the Michaelis constant.

### Detection of the reactive hydroxyl radicals ·OH production

The hydroxyl radicals (·OH) production was measured by fluorescence method. The terephthalic acid was used as a fluorescence probe for detection the ·OH from the H_2_O_2_ for Na_10_[α-GeW_9_O_34_], Na_8_H[α-PW_9_O_34_], Na_10_[α-SiW_9_O_34_] and K_10_P_2_W_18_Fe_4_(H_2_O)_2_O_68_ as catalysts. 75 *μ*L of 25 mM TA in NaOH (pH = 13) solution was added into the 3 mL of PBS (pH = 7.4) containing 100 mM H_2_O_2_ and/or 1 mM POMs. After 24 h incubation in the dark, the resulting solution was detected. The fluorescence spectra were obtained with excitation wavelength of 315 nm and the emission spectra were recorded in the wavelength of 425 nm.

### Detection of H_2_O_2_

The detection of H_2_O_2_ was performed according to the following steps: 480 *μ*L of OPD solution (576 *μ*M) or TMB solution (240 *μ*M), 60 *μ*L of POMs (100 *μ*M) and 60 *μ*L H_2_O_2_ (200 mM) with various concentrations were added into 2400 *μ*L of buffer solution, and the total volume of the mixed solution was 3 mL. After reacting 5 min under the optimum conditions, then the UV-Vis spectrophotometer was used to record the absorbance at 450 nm for OPD and 650 nm for TMB.

## Supplementary information

Supplementary Information
