# Supplementary material for: The Intrinsic Enzyme Activities of the Classic Polyoxometalates
Source: Sci Rep. 2019 Oct 16;9:14832. doi: 10.1038/s41598-019-50539-9 (PMC6795894; doi:10.1038/s41598-019-50539-9)
Supplement: Supplementary file 1 — Supplementary Information [file 41598_2019_50539_MOESM1_ESM.docx]

**Supporting information**

**The Intrinsic Enzyme Activities of the Classic** **Polyoxometalates**

Boyu Zhang^1^, Mingming Zhao^1^, Yanfei Qi^1,^*, Rui Tian^1^, Boye B. Carter^1^, Hangjin Zou^1^, Chuhan Zhang^1^, Chunyan Wang^1^

^1^School of Public Health, Jilin University, Changchun, Jilin 130021, China. Correspondence and requests for materials should be addressed to Y. Q. (email: qiyanfei@jlu.edu.cn)

**Characterization of POMs.** The POMs were prepared according to the literature and identified by FI-IR spectra, UV-Vis spectra, as shown in Supplementary Fig. S1 and Fig. S2.

The Keggin structures of H_3_PW_12_O_40_, H_4_SiW_12_O_40_, H_4_GeW_12_O_40,_ K_4_GeW_12_O_40，_H_3_PMo_12_O_40_, H_4_SiMo_12_O_40_ and Eu_3_PMo_12_O_40_ consists of a central SiO_4_, PO_4_ or GeO_4_ tetrahedral surrounded by four M_3_O_9_ (M=W, Mo) groups formed by edge-sharing octahedrons. These groups are connected to each other by corner-sharing oxygen atoms. The solid state FTIR spectra of H_3_PW_12_O_40_ [Supplementary Figure S2 (a)], H_4_SiW_12_O_40_ [Supplementary Figure S2 (b)], H_4_GeW_12_O_40_ [Supplementary Figure S2 (c)]_,_ K_4_GeW_12_O_40_ [Supplementary Figure S2 (d)], H_3_PMo_12_O_40_ [Supplementary Figure S2 (m)], H_4_SiMo_12_O_40_ [Supplementary Figure S2 (n)] and Eu_3_PMo_12_O_40_ [Supplementary Figure S2 (o)] showed the similar characteristic asymmetric stretching vibrational peaks in wave number region of 800-1100cm^-1^.^-1^For H_3_PW_12_O_40_, H_3_PMo_12_O_40_ and Eu_3_PMo_12_O_40_, the vibrational stretching of P-O bonds was observed at 1080, 1064 and 1062 cm^-1^, respectively. For H_4_SiW_12_O_40_ and H_4_SiMo_12_O_40_, the vibrational stretching of Si-O bonds was observed at 1157 and 1115 cm^-1^. For H_4_GeW_12_O_40_ and K_4_GeW_12_O_40_, the vibrational stretching of Ge-O bonds was observed at 1064 and 1066 cm^-1^, respectively. The vibration stretching of M (Mo, W)-Od bonds of H_3_PW_12_O_40_, H_4_SiW_12_O_40_, H_4_GeW_12_O_40,_ K_4_GeW_12_O_40_ H_3_PMo_12_O_40_, H_4_SiMo_12_O_40_ and Eu_3_PMo_12_O_40_ were observed at 985, 981, 962, 966, 966, 908 and 960 cm^-1^, respectively. Also, the peaks observed from 400 to 900 cm^-1^ were assigned to the asymmetric vibrational stretching of edge sharing M-O-M bonds.

The Na_8_H[α-PW_9_O_34_] [Supplementary FigureS2 (g)], Na_10_[α-SiW_9_O_34_] [Supplementary Figure S2 (h)], Na_10_[α-GeW_9_O_34_] [Supplementary Figure S2 (i)] and K_8_[γ-SiW_10_O_36_] [Supplementary Figure S2 (j)] are the lacunary-Keggin polyoxotungstates, IR spectra of these POMs showed the similar characteristic asymmetric stretching vibrational peaks. The vibrational stretching of W-O_c/b_-W bonds was observed from 460-906 cm^-1^. The vibration stretching of W-O_d_ bonds of Na_8_H[α-PW_9_O_34_], Na_10_[α-SiW_9_O_34_], Na_10_[α-GeW_9_O_34_] and K_8_[γ-SiW_10_O_36_] were observed at 938, 996, 983 and 960 cm^-1^. Also, PO_4_, SiO_4_(Na_10_[α-SiW_9_O_34_]), GeO_4_ and SiO_4_ (K_8_[γ-SiW_10_O_36_]) stretching mode was observed at 1057, 1046, 777 and 1062 cm^-1^, respectively.

The vanadium-replaced Keggin structure of α-1,2,3-K_6_H[SiW_9_V_3_O_34_] [Supplementary Figure S2 (q)] and H_5_PMo_10_V_2_O_40_ [Supplementary Figure S2(p)] consists of a central SiO_4_ or PO_4_ tetrahedron surrounded by four M_3_O_9_ (M=W, Mo and V) groups formed by edge-sharing octahedrons. These groups are connected to each other by corner-sharing oxygen atoms. The solid state FTIR spectrum of SiW-3, PW-6 and PW-8 showed the similar characteristic asymmetric stretching vibrational peaks in wave number region of 800-1100cm^-1^. For α-1,2,3-K_6_H[SiW_9_V_3_O_34_], the vibrational stretching of Si-O bond was observed at 1108 cm^-1^. For H_5_PMo_10_V_2_O_40_, the vibrational stretching of P-O bonds was observed at 1068cm^-1^. The vibration stretching of M (Mo, W, V)-Od bonds of α-1,2,3 -K_6_H[SiW_9_V_3_O_34_] and H_5_PMo_10_V_2_O_40_ were observed at 972 and 962 cm^-1^, respectively. Also, the peaks observed from 470 to 920 cm^-1^ were assigned to the asymmetric vibrational stretching of edge sharing M-O-M bonds. Compared with those nonmetal substituted keggin type polyoxotungstates H_4_SiW_12_O_40_· xH_2_O and H_3_PW_12_O_40_· xH_2_O, the characteristic asymmetric stretching vibrational peaks have red shift.

The structure of Wells–Dawson H_6_P_2_Mo_18_O_62_ [Supplementary Figure S2 (r)], α-(NH_4_)_6_P_2_W_18_O_62_ [Supplementary Figure S2 (f)] and α-K_6_P_2_W_18_O_62_·14H_2_O [Supplementary Figure S2(e)] involves two half units of a central PO_4_ tetrahedron surrounded by nine MO_6_ (M=Mo, W) octahedral. Therefore, four kinds of oxygen atoms appear in the FT-IR of them. The first is due to P-O_a_ in which the oxygen atom is connected to the tungsten/molybdate atom. The second is M-O_b_-M oxygen bridges (corner-sharing oxygen bridges between different M_3_O_13_ groups), the third is M-O_c_-M oxygen bridges (edge-sharing oxygen bridge within M_3_O_13_ groups), and the last is M-O_d_ terminal oxygen atoms. Therefore, four characteristic bands of H_6_P_2_Mo_18_O_62_, α-(NH_4_)_6_P_2_W_18_O_62_ and α-K_6_P_2_W_18_O_62_·14H_2_O were appeared as *ν*_as_ (M-O_d_, 958, 968and 966 cm^−1^); *ν*_as_ (W-O_b_-W, 910, 915 and 917 cm^−1^); *ν*_as_ (W-O_c_-W, 778, 783 and 785 cm^−1^) and *ν*_as_ (P-O_a_, 1080, 1091 and 1091cm^−1^).

In the polyoxanion Na_10_H_2_W_12_O_42_ [Supplementary Figure S2 (k)], four trimetallic W_3_O_13_ subunits are linked together by sharing corners. The center of polyoxoanion[H_2_W_12_O_42_]^10-^ is occupied by two protons instead of a heteroatom in a tetrahedral environment. The strong peak in 960 cm^-1^ can be assigned to the characteristic vibration band of the W-O_d_ bonds，while the strong peaks in 872 and 783 cm^-1^ are attributed to the *ν*_as_( W-Ob/c-W) stretching vibrations.

The IR spectrum of sandwich-type K_10_P_2_W_18_Fe_4_(H_2_O)_2_O_68_ is in agreement with those in the literature, as shown in Supplementary Figure S2 (I). The characteristic vibration patterns derived from Keggin-type polyoxoanions are observed in 1100-700cm^-1^. The obvious characteristic bands at 956, 884, 811, and 742 cm^-1^ are attributed to W-O_d,_ W-O_b_-W and W-O_c_-W, respectively. The strong absorption peaks at 1051 cm^-1^ are associated with the ν_as_ (P-O_a_).

In all spectra, the peaks show around 1600 and 3400 cm^-1^ which is due to water molecules.

In the UV-vis spectra, the five compounds show similar bands at 260 nm, which are associated with terminal W = O_d_ links for πp-d electronic transitions, and W-O-W characteristic tricentric links with oxygen atom bridged between two addenda atoms. These bands in the spectra correspond to those found in the literatures.


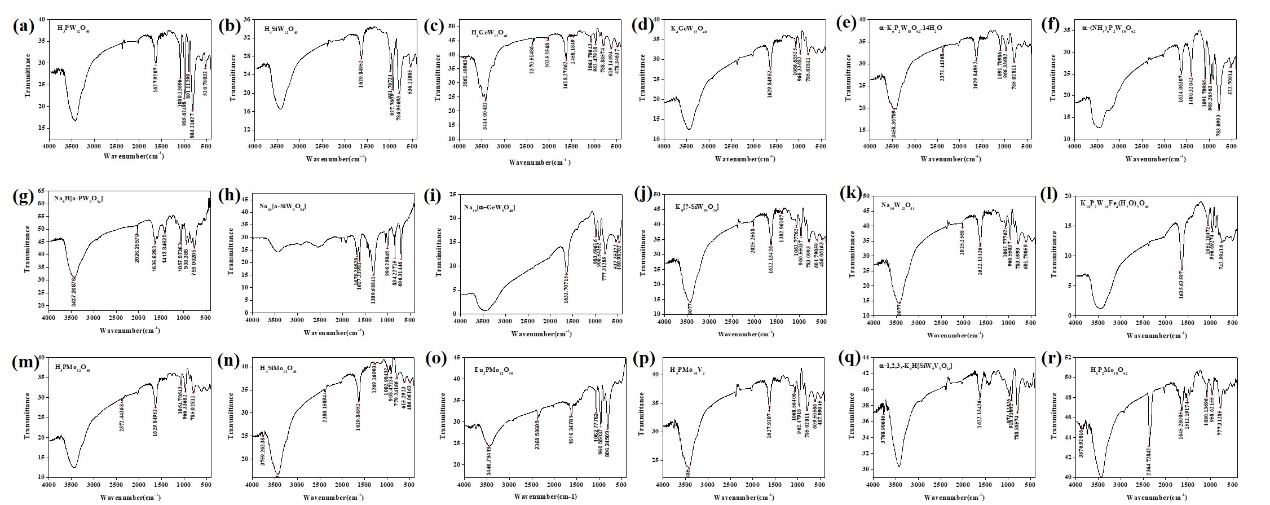


**Figure S1.** IR spectra of POMs. (a) H_3_PW_12_O_40_, (b) H_4_SiW_12_O_40_, (c) H_4_GeW_12_O_40_, (d) K_4_GeW_12_O_40_, (e) α-K_6_P_2_W_18_O_62_·14H_2_O, (f) α-(NH_4_)_6_P_2_W_18_O_62_, (g) Na_8_H[α-PW_9_O_34_], (h) Na_10_[α-SiW_9_O_34_], (i) Na_10_[α-GeW_9_O_34_], (g) K_8_[γ-SiW_10_O_36_], (k) Na_10_H_2_W_12_O_42_, (l) K_10_P_2_W_18_Fe_4_(H_2_O)_2_O_68_), (m) H_3_PMo_12_O_40_, (n) H_4_SiMo_12_O_40_, (o) Eu_3_PMo_12_O_40_, (p) H_5_PMo_10_V_2_O_40_, (q) α-1,2,3, -K_6_H[SiW_9_V_3_O_34_], (r) H_6_P_2_Mo_18_O_62_.


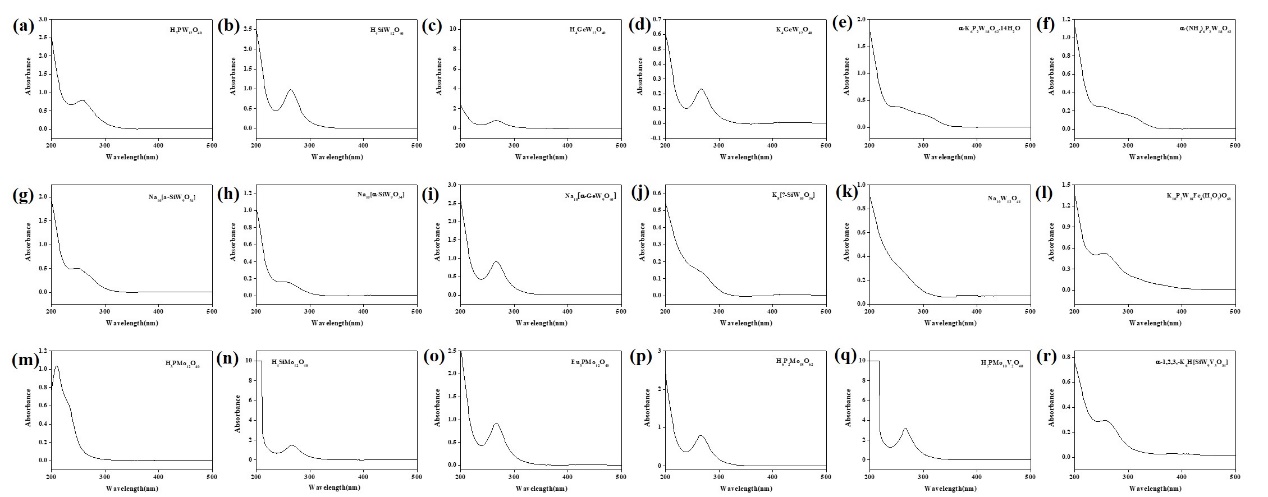


**Figure S2.** UV-Vis spectra of POMs. (a) H_3_PW_12_O_40_, (b) H_4_SiW_12_O_40_, (c) H_4_GeW_12_O_40_, (d) K_4_GeW_12_O_40_, (e) α-K_6_P_2_W_18_O_62_·14H_2_O, (f) α-(NH_4_)_6_P_2_W_18_O_62_, (g) Na_8_H[α-PW_9_O_34_], (h) Na_10_[α-SiW_9_O_34_], (i) Na_10_[α-GeW_9_O_34_], (g) K_8_[γ-SiW_10_O_36_], (k) Na_10_H_2_W_12_O_42_, (l) K_10_P_2_W_18_Fe_4_(H_2_O)_2_O_68_, (m) H_3_PMo_12_O_40_, (n) H_4_SiMo_12_O_40_, (o) Eu_3_PMo_12_O_40_, (p) H_5_PMo_10_V_2_O_40_, (q) α-1,2,3,-K_6_H[SiW_9_V_3_O_34_], (r) H_6_P_2_Mo_18_O_62_.

**
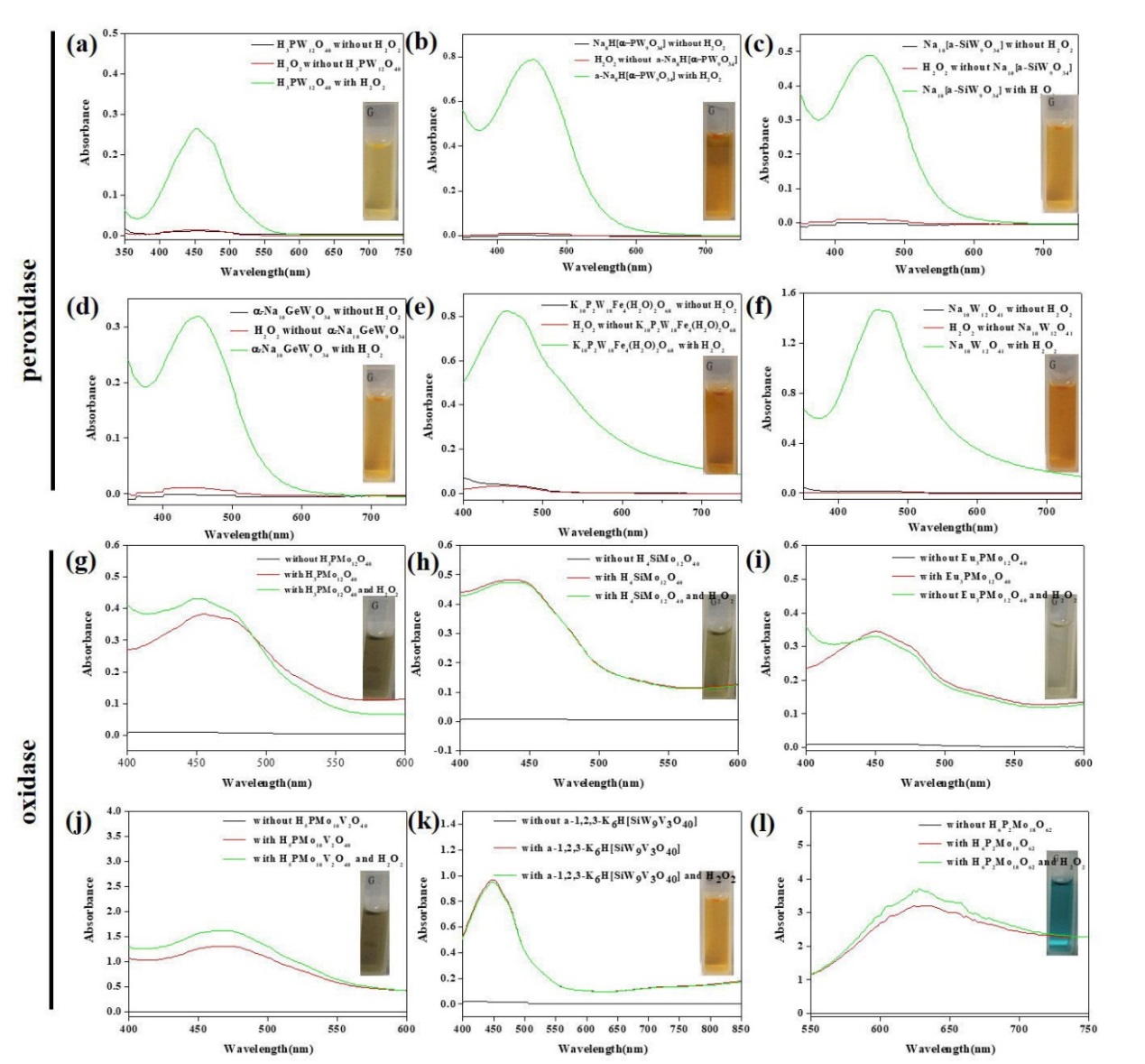
**

**Figure S3****.** The catalytic effect of POMs with OPD as the substrates. (a) H_3_PW_12_O_40_, (b) Na_8_H[α-PW_9_O_34_], (c) Na_10_[α-SiW_9_O_34_], (d) Na_10_[α-GeW_9_O_34_], (e) K_10_P_2_W_18_Fe_4_(H_2_O)_2_O_68_, (f) Na_10_H_2_W_12_O_42_, (g) H_3_PMo_12_O_40_, (h) H_4_SiMo_12_O_40_, (i) Eu_3_PMo_12_O_40_, (g) H_5_PMo_10_V_2_O_40_, (k) α-1,2,3, -K_6_H[SiW_9_V_3_O_34_], (l) H_6_P_2_Mo_18_O_62_ with TMB as the substrate.

**
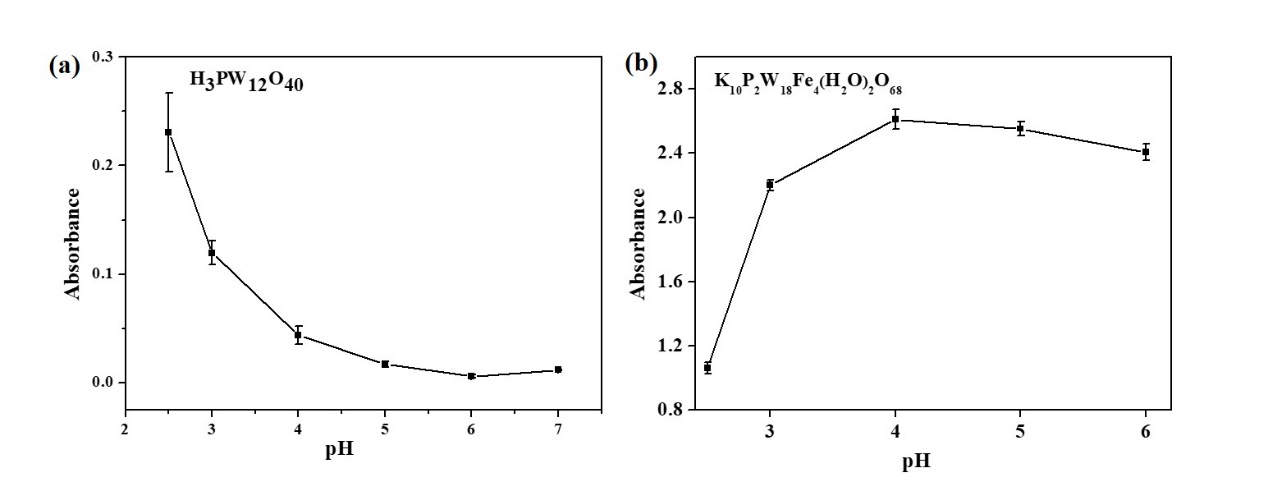
**

**Figure S4.** Effects of pH on peroxidase-like enzymes with TMB as substrates. (a) H_3_PW_12_O_40_ ;(b) K_10_P_2_W_18_Fe_4_(H_2_O)_2_O_68_.

**
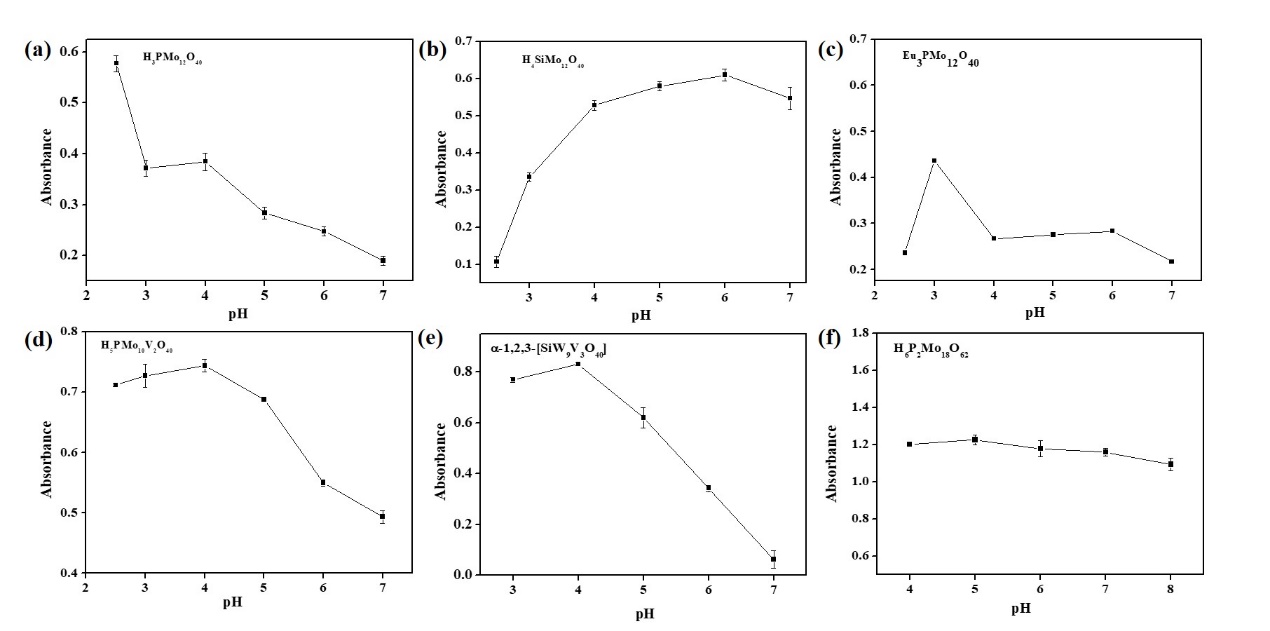
**

**Figure S5.** Effects of pH on oxidase-like enzymes with TMB as substrates. (a) H_3_PMo_12_O_40_, (b) H_4_SiMo_12_O_40_, (c) Eu_3_PMo_12_O_40_, (d) H_5_PMo_10_V_2_O_40_, (e) α-1,2,3- K_6_H[SiW_9_V_3_O_34_], (f) H_6_P_2_Mo_18_O_62_.

**
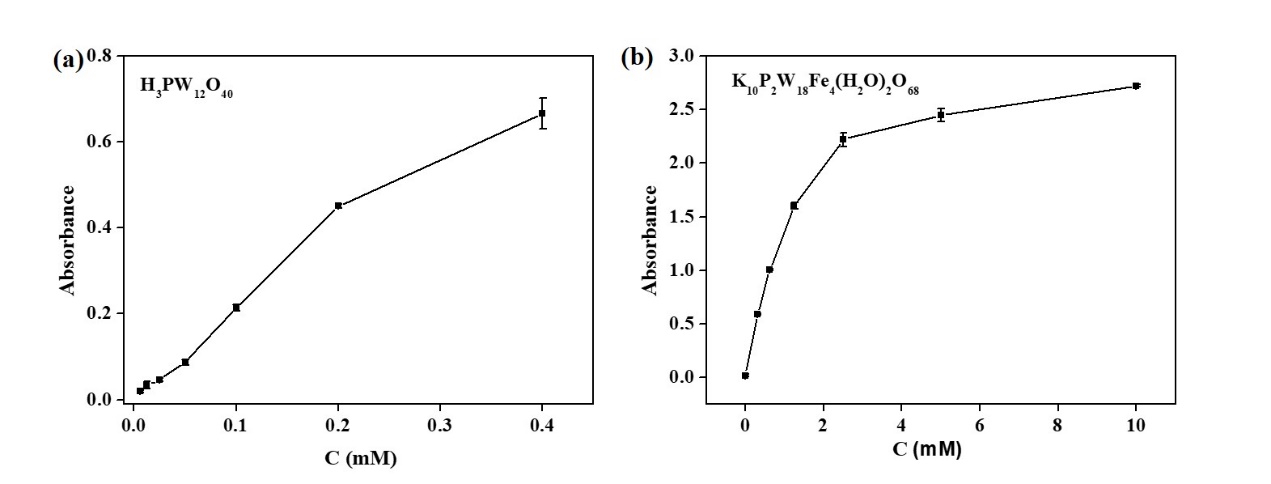
**

**Figure S6.** Effects of concentrations of peroxidase-like enzymes with TMB as substrates. (a) H_3_PW_12_O_40_, (b) K_10_P_2_W_18_Fe_4_(H_2_O)_2_O_68_.

**
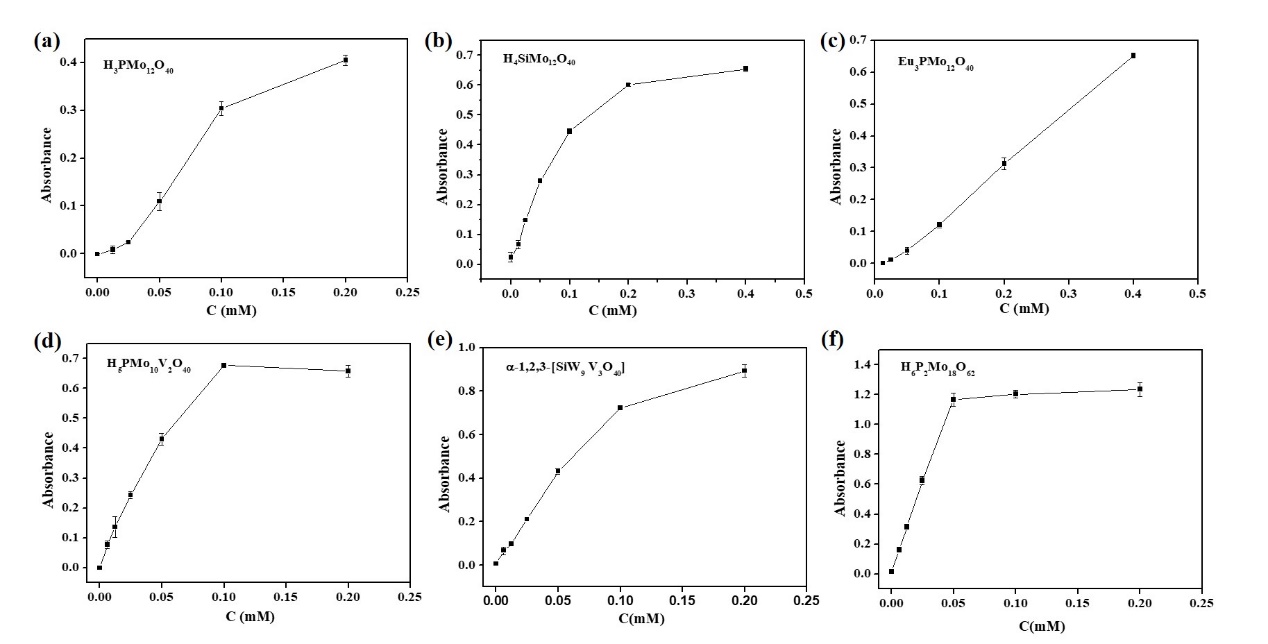
**

**Figure S7.** Effects of concentrations of oxidase-like enzymes with TMB as substrates, (a) H_3_PMo_12_O_40_, (b) H_4_SiMo_12_O_40_, (c) Eu_3_PMo_12_O_40_, (d) H_5_PMo_10_V_2_O_40_, (e) α-1,2,3- K_6_H[SiW_9_V_3_O_34_], (f) H_6_P_2_Mo_18_O_62_.

**
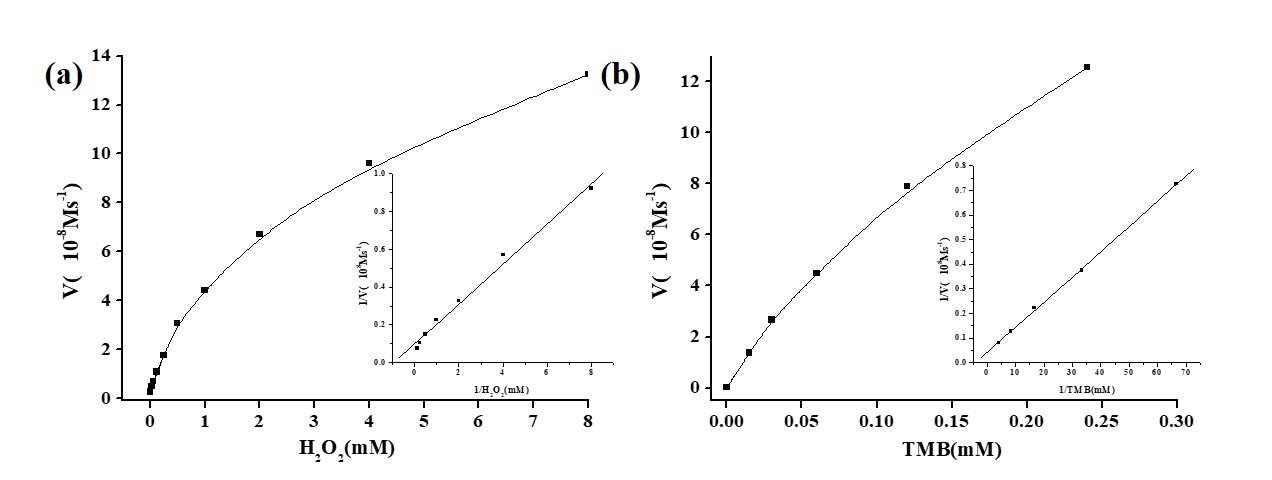
**

**Figure S8.** The steady-state kinetic assay and catalytic mechanism of K_10_P_2_W_18_Fe_4_(H_2_O)_2_O_68_ with TMB as the substrate.

**
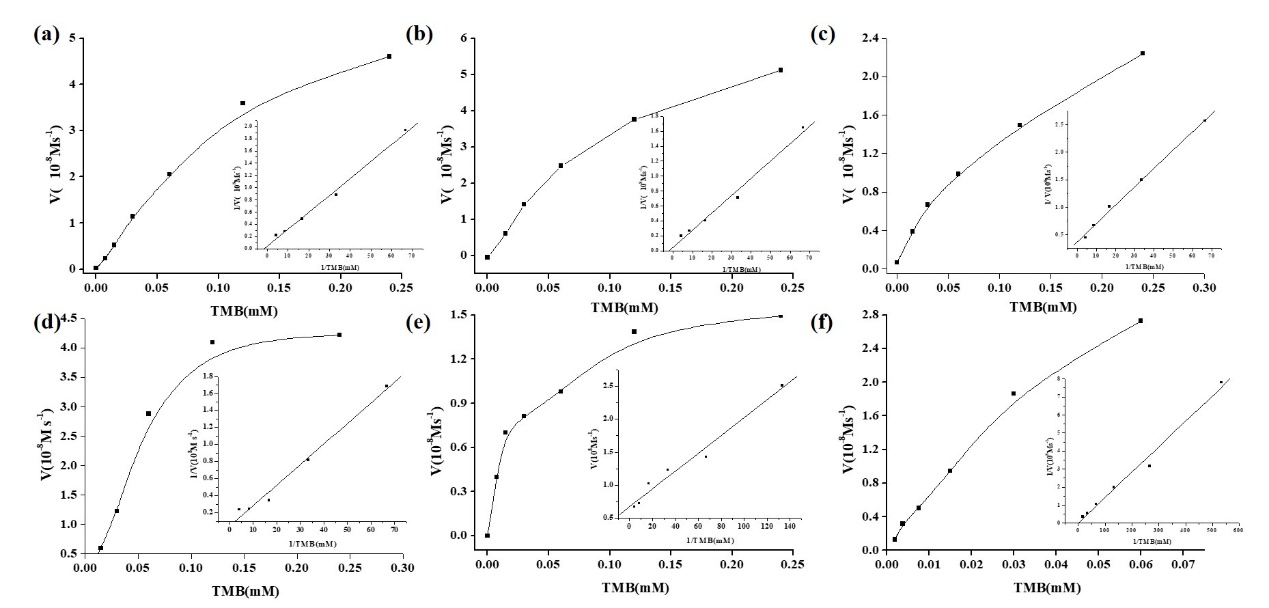
**

**Figure S9.** The steady-state kinetic assay and catalytic mechanism of oxidase-like enzymes with TMB as the substrate. (a)H_3_PMo_12_O_40_, (b)H_4_SiMo_12_O_40_, (c)Eu_3_PMo_12_O_40_, (d) H_5_PMo_10_V_2_O_40_, (e) α-1,2,3- K_6_H[SiW_9_V_3_O_34_], (f) H_6_P_2_Mo_18_O_62_.

**
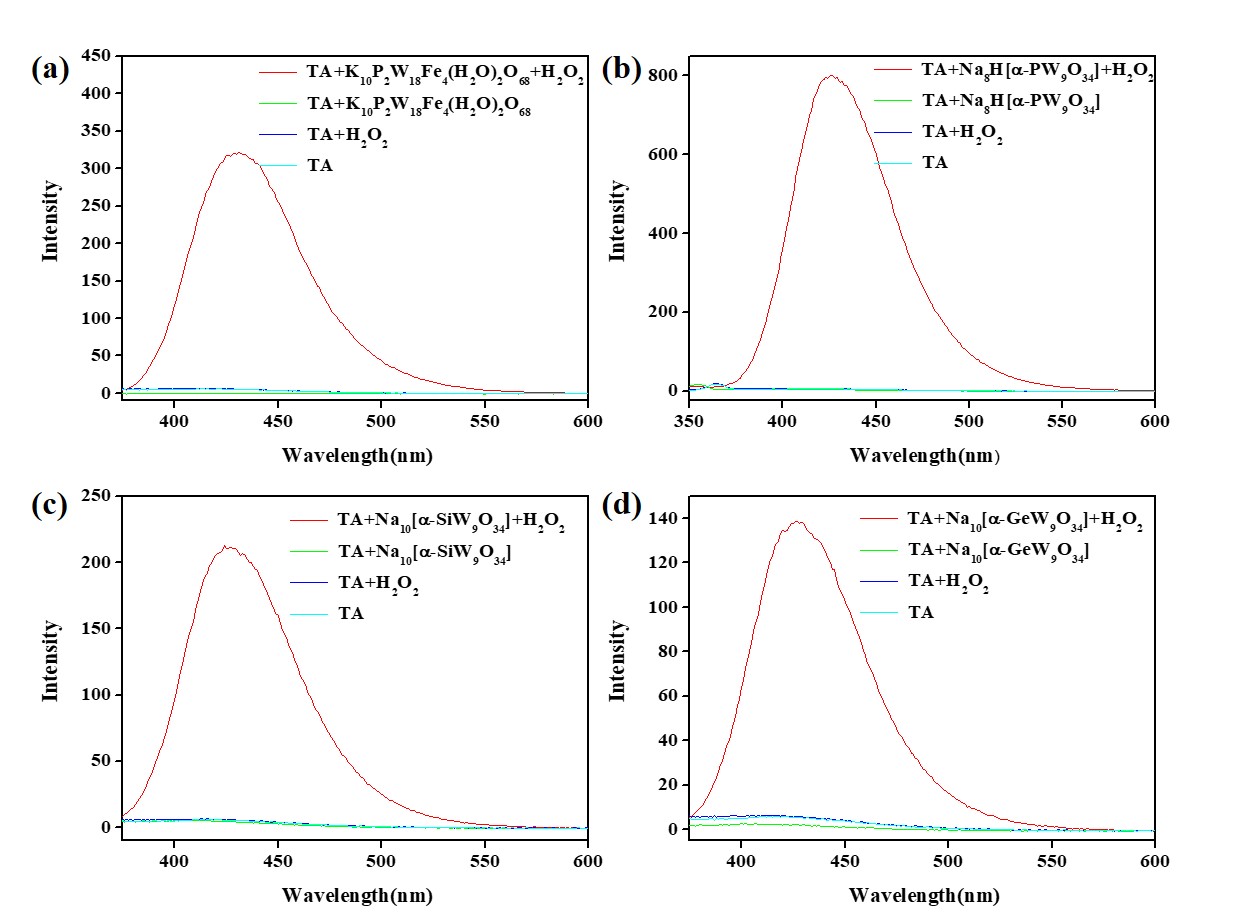
**

**Figure S10.** Fluorescence spectra for detection of hydroxyl radicals ·OH with different controls. (a) K_10_P_2_W_18_Fe_4_(H_2_O)_2_O_68_, (b) Na_8_H[α-PW_9_O_34_], (c) Na_10_[α-SiW_9_O_34_], (d)Na_10_[α-GeW_9_O_34_].

**Table S1** Comparison of the *K_m_* and *V_max_* of peroxidase-like enzymes in TMB system.

| **Nanozymes** | **Substrate** | ***Km* (mM)** | ***Vmax* (M·S^-1^)** | **Reference** |
| --- | --- | --- | --- | --- |
| K_10_P_2_W_18_Fe_4_(H_2_O)_2_O_68_ | TMB | 0.25 | 2.42× 10^-7^ | This work |
| K_10_P_2_W_18_Fe_4_(H_2_O)_2_O_68_ | H_2_O_2_ | 1.09 | 1.02× 10^-7^ | This work |
| FA-Fe_2_SiW_10_ | TMB | 0.014 | 1.42× 10^-7^ | [22] |
| FF@PW_12_@GO | TMB | 0.033 | 10.8×10^-8^ | [27] |
| FF@PW_12_@GO | H_2_O_2_ | 0.214 | 10.5×10^-8^ | [27] |
| C_42_H_118_Bi_2_Cu_7_N_28_Na_4_O_97_W_18_ | TMB | 0.03 | 5.25×10^-8^ | [28] |
| C_42_H_118_Bi_2_Cu_7_N_28_Na_4_O_97_W_18_ | H_2_O_2_ | 0.23 | 7.33×10^-8^ | [28] |
| C_42_H_112_Sb_2_Cu_7_N_28_Na_4_O_94_W_18_ | TMB | 1.24 | 3.02×10^-8^ | [28] |
| C_42_H_112_Sb_2_Cu_7_N_28_Na_4_O_94_W_18_ | H_2_O_2_ | 2.32 | 3.44×10^-8^ | [28] |
| KFePW_12_O_40_ | TMB | 0.346 | 3.7×10^-8^ | [25] |
| KFePW_12_O_40_ | H_2_O_2_ | 165 | 6.9×10^-8^ | [25] |
| Na_2_[V_6_O_13_{(OCH_2_)_3_CCH_2_OH}_2_] | TMB | 0.032 | 2.15×10^-7^ | [31] |
| Na_2_[V_6_O_13_{(OCH_2_)_3_CCH_2_OOCCH_2_CH_3_}_2_] | TMB | 0.026 | 1.91×10^-7^ | [31] |
| Na_2_[V_6_O_13_{(OCH_2_)_3_CCH_2_OOC(CH_2_)_12_CH_3_}_2_] | TMB | 0.021 | 2.09×10^-7^ | [31] |
| HRP | TMB | 0.275 | 1.24×10^−8^ | [21] |
| HRP | H_2_O_2_ | 0.214 | 2.46×10^−8^ | [21] |
